# A freely accessible, adaptable hollow-fiber setup to reproduce first-order absorption: illustration with linezolid cerebrospinal fluid pharmacokinetic data

**DOI:** 10.1128/spectrum.00051-25

**Published:** 2025-05-15

**Authors:** N. Prébonnaud, A. Chauzy, N. Grégoire, C. Dahyot-Fizelier, C. Adier, S. Marchand, V. Aranzana-Climent

**Affiliations:** 1 Université de Poitiers, Inserm U1070, PHAR2, Poitiers, France; 2 Laboratoire de Toxicologie-Pharmacocinétique, CHU de Poitiers 36655, Poitiers, France; 3 Service d’anesthésie-réanimation médecine périopératoire, CHU de Poitiers 36655, Poitiers, France; Innovations Therapeutiques et Resistances, Toulouse, France

**Keywords:** hollow fiber, first-order absorption, pharmacokinetics, antimicrobial agents

## Abstract

The main objective of this study was to validate an algorithm and experimental setup to simulate first-order absorption pharmacokinetic profiles without altering the standard *in vitro* hollow-fiber infection model (HFIM). For that, clinical cerebrospinal fluid (CSF) linezolid concentrations after 30 min infusions at dosing regimens 600 mg q12 h, 900 mg q12 h, and 900 mg q8 h were reproduced in the HFIM over 4 days. To approximate the apparent first-order absorption observed on CSF pharmacokinetic profiles, we split the dosing interval into a series of sub-intervals during which continuous infusions were delivered to the system. During each sub-interval, the same amount of linezolid was delivered, but the sub-intervals had different durations and flow rates, which were computed by a newly developed algorithm. In addition, we independently reproduced plasma concentrations to validate our system. Samples were collected from the central reservoir and the extracapillary space (ECS) of the cartridge of the HFIM and assayed by liquid chromatography-tandem mass spectrometry. Observed pharmacokinetic parameters and concentrations in the ECS were compared with the target clinical pharmacokinetic parameters and concentrations. Observed pharmacokinetic parameters were within 20% of target pharmacokinetic parameters for all experiments, thus validating the ability of our experimental setup to reproduce plasma and CSF linezolid pharmacokinetic profiles. The algorithm and setup are available in the open-source web application (https://varacli.shinyapps.io/hollow_fiber_app/) to easily design other HFIM experiments.

**IMPORTANCE:**

We developed and validated a novel hollow-fiber setup that enables *in vitro* simulation of mono-compartmental pharmacokinetics with an absorption phase. This novel experimental setup was easily implemented on top of the traditional one since it only requires the addition of a programmable infusion pump. Using this streamlined approach, we successfully replicated pharmacokinetics at the infection site, specifically cerebrospinal fluid concentrations of linezolid, consistent with those observed in intensive care unit patients. Thus, this study addresses the challenge of accurately reproducing target site concentrations, rather than relying solely on plasma levels, offering a valuable tool for optimizing dosing regimens in antibiotic therapy. Importantly, this setup also allows for the reproduction of plasma pharmacokinetics following oral (or any other extravascular) administration, broadening its applicability. The algorithm and setup developed in this study were incorporated into an open-source web application designed to facilitate the design of hollow-fiber experimental protocols (https://varacli.shinyapps.io/hollow_fiber_app/).

## INTRODUCTION

The hollow-fiber infection model (HFIM) is a preferential model of *in vitro* pharmacokinetic/pharmacodynamic (PK/PD) study to predict bacterial killing induced by various PK profiles in order to optimize dosing regimen (1). Traditionally, PK/PD indices are determined by performing dose-fractionation studies in mice (2). However, PK observed in animals can be different compared to that in humans. For instance, drug half-life is usually faster in mice than in humans (3). Thus, HFIM is interesting because it can reproduce the PK observed in humans and over a longer duration than in *in vivo* studies, which are generally limited to 24 h (1).

Most HFIM studies reproduced unbound plasma PK profiles observed after intravenous administration and were most often characterized by one- or two-compartment models with first-order elimination and no absorption (4–9). Unbound plasma concentrations are considered the best surrogate of the concentrations observed at infection sites (10). However, the antibiotic concentration at the infection site may be lower than the unbound plasma concentrations depending on the physicochemical properties of the antibiotic and the presence of anatomical barriers (11).

This is particularly true in the context of treating cerebral infections. The brain is protected by the blood-brain barrier and the blood-cerebrospinal fluid (CSF) barrier, which limit the distribution of antibiotics in the cerebrospinal fluid (12). Although meningeal inflammation can increase the permeability of these barriers, antibiotic concentrations in CSF often remain lower and delayed when compared with those in plasma (12, 13). Thus, using plasma concentrations to optimize the dosing regimen can overestimate the effect at the infection site (e.g., CSF), making it more relevant to reproduce concentrations observed at the infection sites in the HFIM to optimize the dosing regimen.

Studies reproducing concentrations observed at infection sites (e.g., CSF concentrations and lung tissue concentrations) are rare in the literature (14–16). In the studies conducted by Hope et al. and Kloprogge et al., the concentration profiles at infection sites reproduced in the HFIM were similar to those observed in plasma, which allowed them to set the programmable syringe to deliver a short infusion, as it is typically done to reproduce plasma concentrations (15, 16).

However, PK profiles observed at infection sites can differ from those observed in plasma due to the presence of an ascending phase that is often slower and delayed when compared with the one observed in plasma, which cannot be mimicked by a short infusion. A way to reproduce PK profiles with absorption is to add a bottle in the HFIM to mimic a depot compartment (17). However, it consumes materials and additional medium, increasing the cost of the experiment.

Moreover, the reproduction of clinical concentrations in the HFIM is not always clearly demonstrated in certain studies (14, 15, 18–21).

In this context, the main objective of this study was to validate an experimental setup to simulate first-order absorption PK profiles without additional compartment in an *in vitro* HFIM. Secondarily, we also wanted to validate the ability of our system to reproduce intravenous PK profiles. For this purpose, CSF and plasma concentrations of linezolid from a previous study on intensive care unit (ICU) patients (13) after three dosing regimens were reproduced in an HFIM.

## MATERIALS AND METHODS

**Fig 1:**
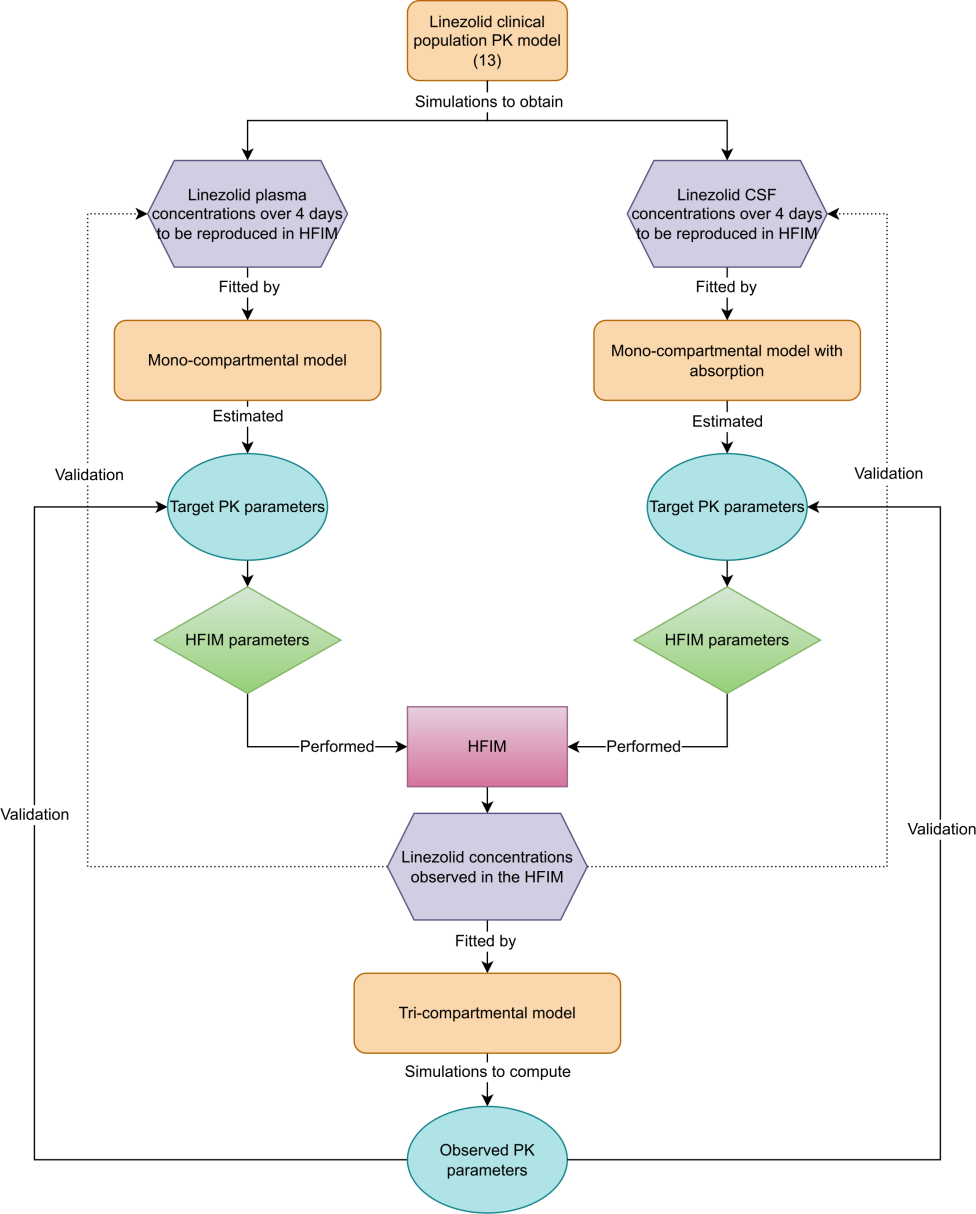
Diagram of the methods used in this study to reproduce either plasma or CSF concentrations in an HFIM (13). Linezolid clinical population PK model from Dahyot-Fizelier et al. Orange boxes correspond to models used, gray boxes represent linezolid concentrations, blue ovals correspond to PK parameters (e.g., *k*_*e*_ and *C*_max_), green lozenges correspond to parameters obtained from the R-shiny application HF-App (i.e., pump flow rates, infusion solution concentration, and volume of diluent), and the pink box represents the *in vitro* HFIM. Created in drawio (22).

A graphical summary of the methods used in this study is presented in Fig. 1.

### Linezolid PK simulations

To generate unbound linezolid concentrations in plasma and CSF from ICU patients, we used the typical profile obtained from the linezolid clinical population PK model developed in a previous study (13), which aimed to evaluate the distribution of this antibiotic in the CSF of ICU patients with external ventricular drainage. The linezolid clinical population PK model consisted of a bi-compartmental model, with one compartment representing plasma concentrations, one compartment representing CSF concentrations, and exchange between these two compartments. Linezolid unbound plasma and CSF concentrations to be reproduced in the HFIM were simulated over 4 days for two standard dosing regimens: 30 min infusion of 600 mg every 12 h, 30 min infusion of 900 mg every 12 h, and one high dosing regimen 30 min infusion of 900 mg every 8 h. Simulations were performed using the mrgsolve R package (23) with R software, version 4.2.2 (24).

### Estimation of target PK parameters to reproduce plasma concentrations in HFIM

Typical profiles of linezolid unbound plasma concentrations, following an infusion (*t*_infusion_) of 0.5 h at the three dosing regimens were approximated with a mono-compartmental model to compute the target elimination rate constant (*k*_*e*_), maximal concentration after the first dose (*C*_max,1_), and concentration at steady state (*C*_max,ss_) using R software, version 4.2.2 (24). This approximation, using a mono-compartmental model while simulated plasma concentrations were obtained with a bi-compartmental model, did not induce significant difference between the parameters obtained by the two methods and enabled plasma concentrations to be easily reproduced in HFIM. From the target *k*_*e*_ value, terminal half-life (*t*_1/2_) was computed as well as the number of doses needed to reach steady state (*N*_dose_), considering 3.3 half-lives to reach 90% of the steady state. The accumulation factor (Rac) was computed from the target *C*_max,ss_ divided by target *C*_max,1_ values. Parameters are given in Table 1.

**TABLE 1:** Target PK parameters for unbound plasma concentrations

Parameter	Dosing regimen		
	600 mg q12 h	900 mg q12 h	900 mg q8 h
*C*_max,1_ (mg/L)	10.7	16.1	16.1
*C*_max,ss_ (mg/L)	11.5	17.2	19.2
Rac	1.07	1.07	1.19
*k*_*e*_ (h^−1^)	0.230		
*t*_1/2_ (h)	3.01		
*N*_dose_	1		2
*t*_infusion_ (h)	0.500		

### Estimation of target PK parameters to reproduce CSF concentrations in HFIM

Typical profiles of linezolid CSF concentrations were approximated by a mono-compartmental model with first-order absorption to compute the absorption rate constant (*k*_*a*_), elimination rate constant (*k*_*e*_), maximal concentration after the first dose (*C*_max,1_), concentration at steady state (*C*_max,ss_), time to reach *C*_max_ after the first dose (*T*_max,1_), and at steady state (*T*_max,ss_) using R software, version 4.2.2 (24). As we made an approximation using a mono-compartmental model while the simulated CSF concentrations were obtained with a bi-compartmental model, the rate constants were empirically adjusted to yield a visually better match between concentrations predicted by the mono-compartmental model and concentrations from the linezolid clinical population PK model (13). From the target *k*_*e*_ value, terminal half-life (*t*_1/2_) was computed as well as the number of doses needed to reach steady state (*N*_dose_), considering 3.3 half-lives to reach 90% of the steady state. The accumulation factor (Rac) was computed from the target *C*_max,ss_ divided by target *C*_max,1_ values. Parameters are given in Table 2.

**TABLE 2:** Target PK parameters for CSF concentrations

Parameter	Dosing regimen		
	600 mg q12 h	900 mg q12 h	900 mg q8 h
*C*_max,1_ (mg/L)	4.25	6.38	6.38
*T*_max,1_ (h)	4.10		
*C*_max,ss_ (mg/L)	5.16	7.73	9.83
*T*_max,ss_ (h)	27.6		27.0
Rac	1.21	1.21	1.54
*k*_*a*_ (h^−1^)	0.300	0.300	0.300
*k*_*e*_ (h^−1^)	0.230	0.230	0.190
*t*_1/2_ (h)	3.01	3.01	3.65
*N*_dose_	1	1	2

### Hollow-fiber infection model

#### General overview of the HFIM

**Fig 2:**
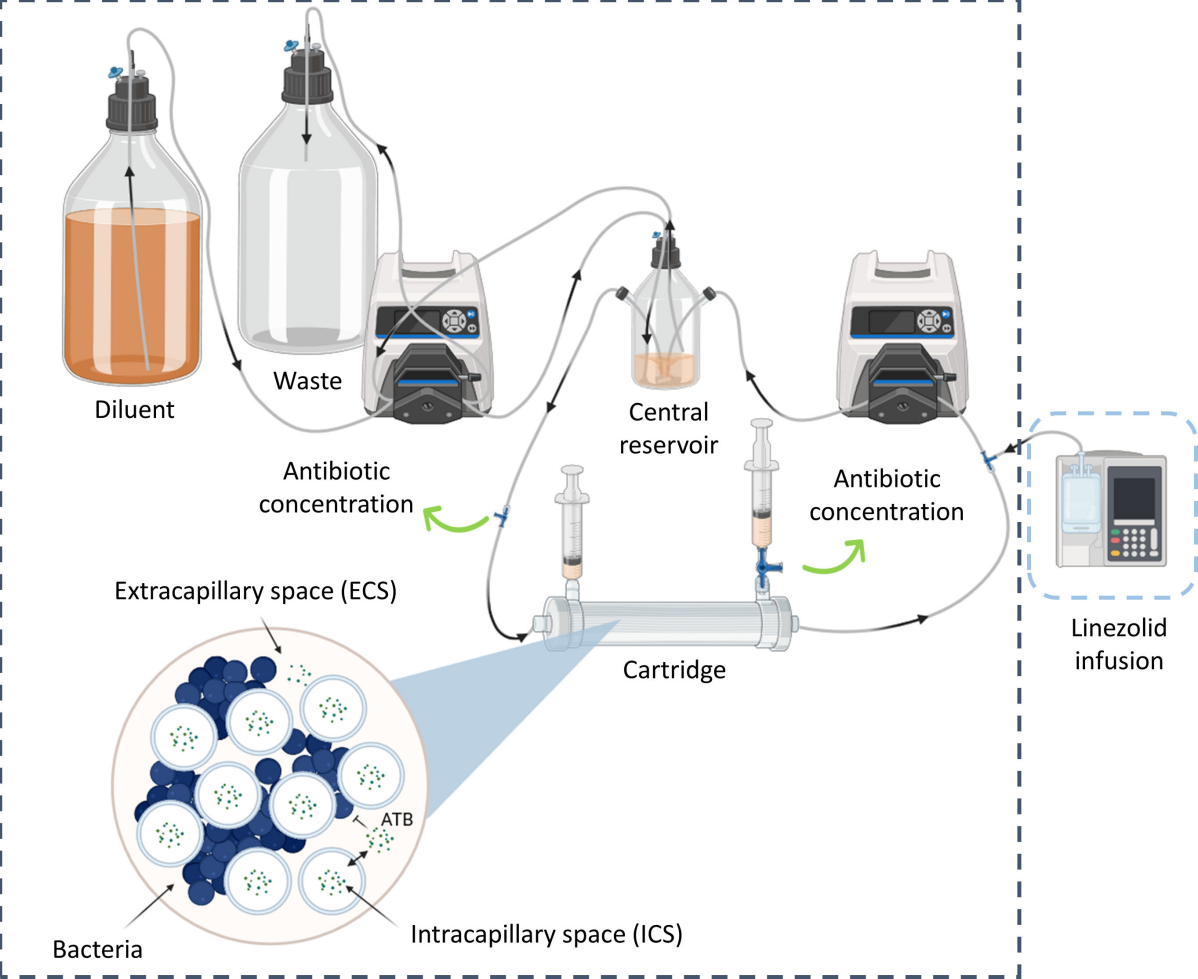
Diagram of hollow-fiber infection model. Black arrows correspond to the direction of flow. Green arrows correspond to sampling sites. Gray dashed line corresponds to the incubator at 37°C ± 2°C. Blue dashed line corresponds to the refrigerated box (2°C–10°C). Created in BioRender.com.

Linezolid unbound plasma and CSF concentrations after 30 min infusion of 600 mg every 12 h, 30 min infusion of 900 mg every 12 h, and 30 min infusion of 900 mg every 8 h were reproduced in the HFIM at 37°C ± 2°C for 4 days at least in duplicate on separate occasions for each dosing regimen. A diagram of the HFIM is presented in Fig. 2.

At the beginning of each experiment, stock solution of linezolid (purity >99% in powder form, Sigma-Aldrich, Merck KGaA, Saint-Quentin-Fallavier, France) at 10,000 µg/mL in dimethyl sulfoxide (DMSO, Merck KGaA, Saint-Quentin-Fallavier, France) was thawed from the freezer at −80°C. Then, a linezolid infusion bag was freshly prepared by diluting stock solution to the desired concentration in 0.9% sodium chloride (Merck KGaA, Saint-Quentin-Fallavier, France). The concentration of the infusion solution as well as the DMSO percentage (< 13%) depended on the dosing regimen (Tables S1 and S2). Then, the infusion bag was placed into an ambulatory infusion pump (Mini Rythmic Perf+, Micrel Medical Devices S.A., Koropi, Athens, Greece), which was kept in a refrigerated box (2°C–10°C) throughout the experiment (Fig. 2). The ambulatory infusion pump delivered the linezolid into a fast-flowing circulation loop connecting the central reservoir containing 300 mL cation-adjusted Mueller-Hinton broth (CAMHB, leading to a final DMSO concentration lower than 0.2%) to the intracapillary space (ICS) of a dialysis cartridge (FX paed helixone dialyzer, Fresenius Medical Care, Bad Homburg, Germany) via a peristaltic pump (Masterflex L/S, Cole Parmer, Roissy, France). The flow rate in the circulation loop was set to 60 mL/min in order to obtain a rapid equilibration of linezolid concentrations between the central reservoir and the ICS of the cartridge. Extracapillary space (ECS) of the cartridge was filled with 60 mL of cation-adjusted Mueller-Hinton broth (Merck KGaA, Saint-Quentin-Fallavier, France) and inoculated by *Staphylococcus aureus* American Type Culture Collection 29213 (Fig. 2).

The hollow fibers were semi-permeable polysulfone membranes with pore size varying between 1.8 and 3.3 nm, which allowed diffusion of linezolid between the ICS and the ECS, whereas bacteria were retained in the ECS (Fig. 2).

To mimic the elimination of linezolid, a second peristaltic pump added antibiotic-free CAMHB (diluent) to the central reservoir and removed excess CAMHB (waste), thus diluting linezolid while maintaining a constant volume of 300 mL of CAMHB in the central reservoir (Fig. 2).

#### Setup to reproduce linezolid plasma concentrations in HFIM

To reproduce linezolid unbound plasma concentrations in the HFIM, the ambulatory pump was set to deliver an infusion of 5 mL over 30 min every 12 h, or 8 h in the case of 900 mg q8 h. The central reservoir volume, flow rate from the central reservoir to the cartridge, cartridge volume, infusion duration and volume to be infused for one dose, dosing interval, total number of doses, experiment duration, target maximal concentration after the first dose (*C*_max,1_), and terminal half-life (*t*_1/2_) defined in Table 1 were entered into the R-shiny application HF-App (25) to obtain the experimental parameters (flow rate from the central reservoir to the waste, diluent volume, and infusion solution concentration) given in Table S1.

#### Setup to reproduce linezolid CSF concentrations in HFIM

To reproduce linezolid CSF concentrations in the HFIM, we approximated the apparent first-order absorption by a series of continuous infusions. To do so, we divided each dosing interval into 12 sub-intervals (or 8 sub-intervals in the case of 900 mg q8 h). Over all sub-intervals, the same amount of linezolid was administered; only the duration and rate of administration differed between each sub-interval.

For a given target PK profile, the duration and infusion flow rates for each sub-interval were computed using the following algorithm (details about the algorithm construction are provided in Text S1):

1. The target maximal concentration after the first dose (*C*_max, 1_ in mg/L), absorption rate constant (*k*_*a*_ in h^−1^), the elimination rate constant (*k*_*e*_ in h^−1^), terminal half-life (*t*_1/2_ in h), and the end time of the last sub-interval (*t*_*n*_ in h) were chosen based on the target PK profile to be simulated (Table 2; Table S2).
2. The central reservoir and hollow-fiber cartridge volume (*V*_total_ in L) were chosen based on what was most practical in our experimental system. The number of sub-intervals (n) was chosen based on the capabilities of our programmable ambulatory infusion pump (Table S2).
3. The time to reach *C*_max,1_ was computed using equation 1.

(1) $$t_{max,1}=\frac{1}{k_{a}-k_{e}}ln\left(\frac{k_{a}}{k_{e}}\right)$$

- The dose administered at the end of the last sub-interval (Dose) with bioavailability (*F*) was fixed to 1, and the fraction of target dose administered at the end of the last sub-interval (*f*_dose_) was computed using the equations (equations 2 and 3) below.

(2) $$Dose=C_{max,1}\times {\left[\frac{F\;\times \;k_{a}}{V_{total}\times \left(k_{a}-k_{e}\right)}\left(e^{-k_{e}\times t_{max,1}}-e^{-k_{a}\times t_{max,1}}\right)\right]}^{-1}$$

(3) $$f_{dose}=1-\;e^{-k_{a}\;\times \;t_{n}}$$

For each sub-interval *i*, *t*_*i*_ (h)—the end time of sub-interval, *A*_*i*_ (mg)—the amount of drug remaining to be administered for each *t*_*i*_, and *S*_*i*_ (mg/h)—the infusion rate were computed using the equations (equations 4 to 6) below.

(4) $$t_{i}=-\frac{ln\left(1-f_{dose}\frac{i}{n}\right)}{k_{a}}$$

(5) $$A_{i}=Dose\times e^{-k_{a}\times t_{i}}$$

(6) $$S_{i}=\frac{A_{i-1}-A_{i}}{t_{i}-t_{i-1}}$$

- The linezolid concentration of the infused solution (*C*_infusion_) was chosen so that the volume administered during each sub-interval was as close as possible to 2 mL (lowest possible injectable volume with good precision with our ambulatory infusion pump) (Table S2).
- For each sub-interval, the infusion pump flow rate (Flow_infusion,*i*_) was computed using equation 7 below:

(7) $$Flow_{infusion,i}=\frac{S_{i}}{C_{infusion}}$$

- Infusion pump flow rates were then manually adjusted to the closest value programmable in the ambulatory infusion pump.

The flow of the pump adding diluent to the central reservoir (CL_elim_) was computed with equation 8 (Table S2).


(8) $$CL_{elim}=k_{e}\times V_{total}$$

Diluent volume (*V*_diluent_) was then computed with equation 9 for an experiment duration (Exp_duration_) of 96 h (Table S2).


(9) $$V_{diluent}=CL_{elim}\times Exp_{duration}$$

This algorithm is implemented in the freely accessible R-shiny application HF-App (25), and all experimental parameters used are summarized in Table S2.

To evaluate the ability of the algorithm to approximate mono-compartmental kinetics with first-order absorption, simulations of the target PK profiles using a mono-compartmental model with first-order absorption (with parameters from Table 2) and simulations of expected concentrations with the infusions computed with the algorithm (settings found in Table 4 and Table S3) were performed. Simulations were performed using the mrgsolve R package (23) with R software, version 4.2.2 (24). These were compared by computation of mean percentage error to evaluate bias and root mean squared error to evaluate imprecision.

To ensure that linezolid unbound plasma and CSF concentrations were well reproduced in the HFIM, serial samples were collected from the central reservoir and the ECS of the cartridge at various time points. Samples from the ECS of the cartridge were centrifuged at 13,000 rpm, and only the supernatant without bacteria was kept. Samples were stored at −80°C until assayed by liquid chromatography coupled with tandem mass spectrometry (LC-MS/MS).

### LC-MS/MS assay

The analysis of linezolid concentrations in CAMHB was performed by an LC-MS/MS method previously developed for assay of linezolid plasma concentrations (13). Calibration curves were established over 0.2–80 µg/mL. Samples were precipitated with 300 µL of acetonitrile containing the internal standard (Linezolid D8, purity of 100% in powder form, Alsachim, Illkirch Graffenstaden, France) at 300 ng/mL and vortexed for 10 s. Then, samples were centrifuged at 14,000 rpm for 20 min at 4°C, and 100 µL of supernatant was transferred into a glass vial containing 100 µL of 10 mM ammonium formate. A volume of 2 µL was injected.

The system included a Shimadzu high-performance liquid chromatography system module (Nexera XR; Shimadzu, Marne la Vallée, France) coupled with a TQ3500 mass spectrometer (Sciex, Les Ulis, France). The compound was analyzed on an XBridge Peptide BEH300 C18 column (5 µm, 2.1 × 150 mm, Waters, Saint-Quentin-en-Yvelines, France). The mobile phase consisted of a mixture (50/50, vol/vol) of 10 mM ammonium formate and acetonitrile delivered isocratically at 0.20 mL/min. Electrospray ionization in positive mode was used for detection. Ions were analyzed in the multiple reaction monitoring, and the following transitions were inspected: m/z 338→296 and 346→304 for linezolid and internal standard, respectively.

The intraday variability was characterized at three concentration levels (0.6, 15, and 60 µg/mL) with a precision <3% and bias <9%. The interday variability was characterized at three concentration levels (0.6, 15, and 60 µg/mL) with a precision <5% and bias <2% (*n* = 19).

### Modeling of observed concentrations in the HFIM reproducing plasma and CSF linezolid PK

#### Model structure

**Fig 3:**
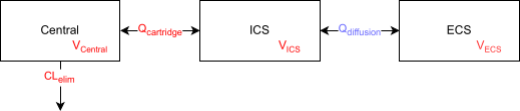
Structure of tri-compartmental model of HFIM. Central corresponds to the central reservoir of the HFIM, ICS to the intracapillary space of the cartridge, ECS to the extracapillary space of the cartridge, CL_elim_ to the pump flow rate from the central reservoir to the waste, *Q*_cartridge_ to the pump flow rate from the central reservoir to the cartridge, and *Q*_diffusion_ to the diffusion flow rate between ICS and ECS of the cartridge. Red parameters were fixed, and the blue parameter was estimated.

Linezolid concentrations observed in the central reservoir and ECS of the cartridge of the HFIM were analyzed simultaneously. A tri-compartmental model (Fig. 3), based on the structure of the experimental hollow fiber, was used to fit the data to account for a possible delay in linezolid concentrations in the ECS due to the diffusion between the ICS and ECS of the cartridge. Although it is assumed that the concentrations between the central reservoir and the cartridge equilibrate rapidly due to the high flow rate of the pump between the central reservoir and the cartridge, this has not been demonstrated (26, 27).

#### Estimation of constant diffusion flow rate between ICS and ECS

The tri-compartmental model allowed us to estimate the constant diffusion flow rate between ICS and ECS of the cartridge (*Q*_diffusion_ in L/h) using Monolix (Monolix 2024R1, Lixoft SAS, a Simulations Plus company). To do so, the values of the central reservoir volume (*V*_Central_ in L), pump flow rate from the central reservoir to the waste (CL_elim_ in L/h), pump flow rate from the central reservoir to the cartridge (*Q*_cartridge_ in L/h), and the cartridge volume (*V*_cartridge_ in L) were fixed to experimental values (Tables S1 and S2). Volume of ICS (*V*_ICS_ in L) was computed by equation 9 using the fraction of cartridge volume taken by fibers (*F*_ICS_, determined from the cartridge technical information given in Text S2) and the cartridge volume (*V*_cartridge_ in L). Volume of ECS (*V*_ECS_, in L) was computed using equation 10.


(9) $$V_{ICS}=F_{ICS}\times V_{cartridge}$$

(10) $$V_{ECS}=V_{cartridge}-V_{ICS}$$

#### Validation of the tri-compartmental model

Model fit to data was evaluated by performing 1,000 simulations with residual unexplained variability from the final model and plotting observed data against the 90% prediction interval. The model was considered valid when 90% of observed data was within the 90% prediction interval.

#### Validation of the HFIM setup

For each dosing regimen, the observed PK parameters in the ECS of the cartridge of the HFIM were computed by simulation under the final tri-compartmental model using the mrgsolve R package (23) with R software, version 4.2.2 (24). Computed observed PK parameters include the following:

- Maximal concentration after the first dose (*C*_max,1_) and at steady state (*C*_max,ss_)
- Time to reach *C*_max_ after the first dose (*T*_max,1_) and at steady state (*T*_max,ss_)
- Area under the curve for a dosing interval after the first dose (AUC_τ, 1_) and at steady state (AUC_τ, ss_)
- Elimination half-life (*t*_1/2_)

Our setup to reproduce plasma or CSF concentrations was considered valid when the computed observed PK parameters in the ECS of the cartridge were within 20% of the target PK parameters.

## RESULTS

### Tri-compartmental model fits well the observed concentrations in the HFIM reproducing plasma and CSF linezolid PK

**Fig 4:**
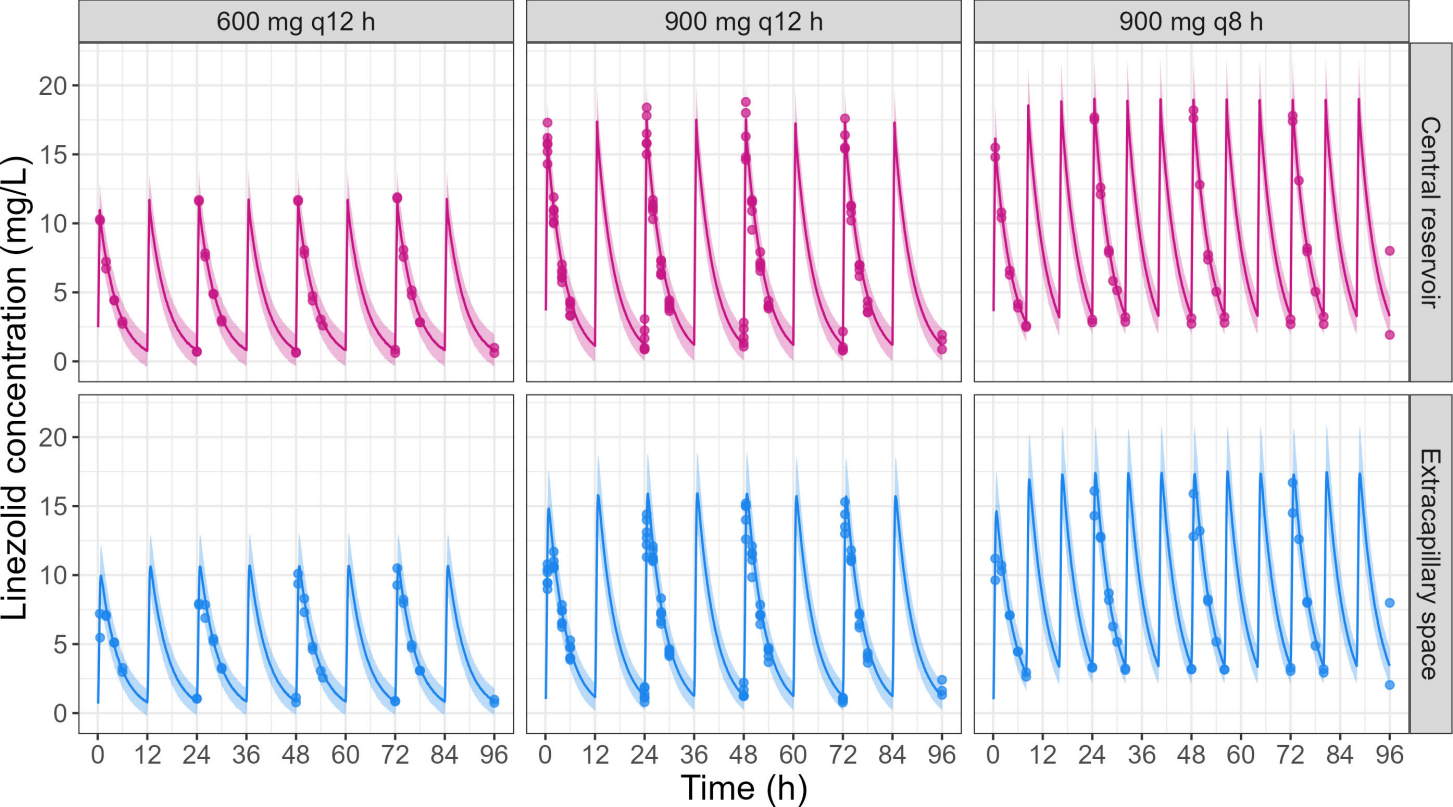
Observations vs simulations under the final model of linezolid concentrations observed in the central reservoir and in the cartridge ECS of the HFIM reproducing plasma PK for the three tested dosing regimens (replicate number = 2–3). Points correspond to the observed linezolid concentrations in HFIM. The line corresponds to the median of the simulations. The colored area corresponds to the 90% prediction interval (i.e., constructed from simulations with residual unexplained variability).

**Fig 5:**
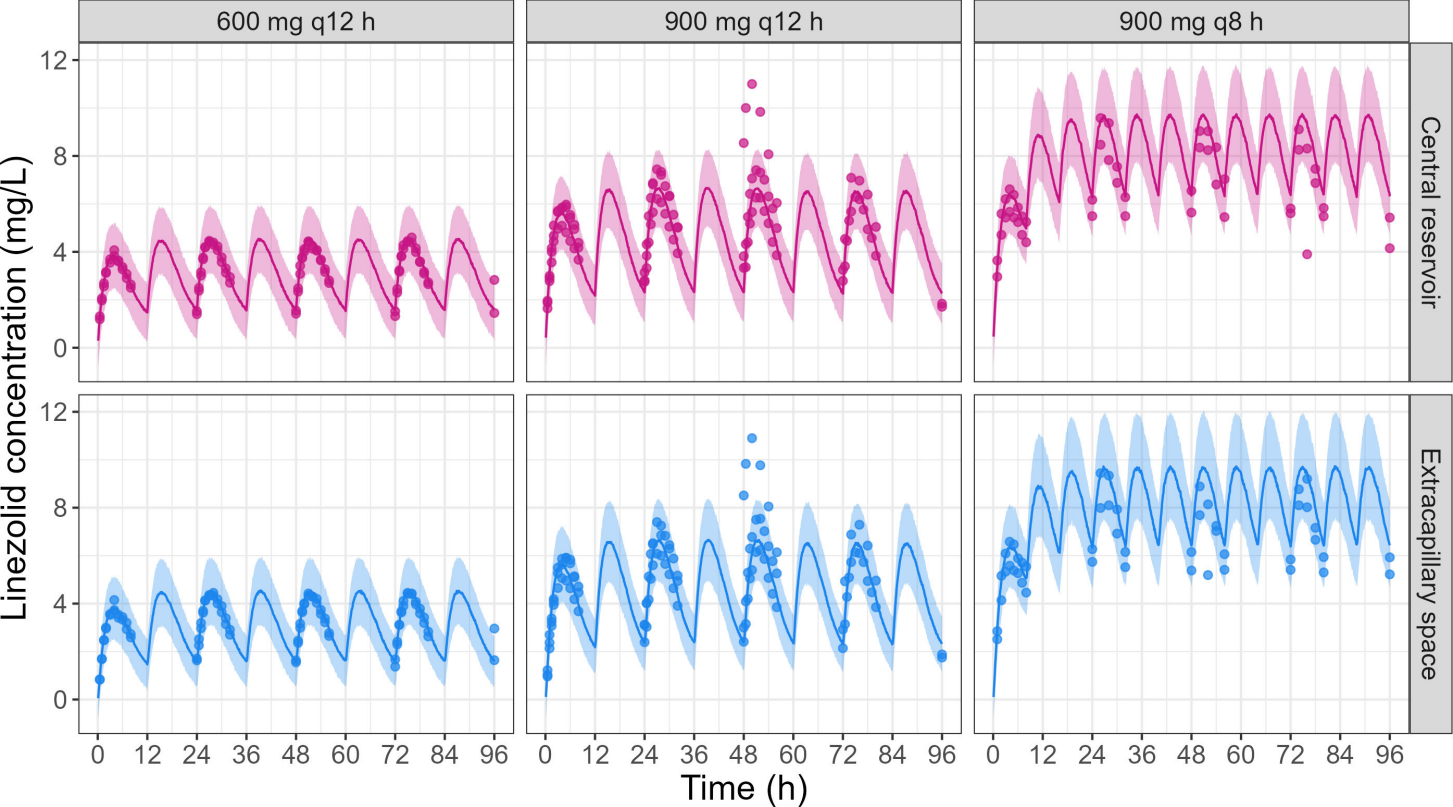
Observations vs simulations under the final model of linezolid concentrations observed in the central reservoir and in the cartridge ECS of the HFIM reproducing CSF PK for the three tested dosing regimens (replicate number = 2–3). Points correspond to the observed linezolid concentrations in HFIM. The line corresponds to the median of the simulations. The colored area corresponds to the 90% prediction interval (i.e., constructed from simulations with residual unexplained variability).

The tri-compartmental model fitted linezolid concentrations well, for the setup mimicking plasma (Fig. 4) and CSF PK (Fig. 5) for both the central reservoir and the ECS of the hollow-fiber cartridge.

### Diffusion across hollow-fiber membrane shows an apparent lag time

**Fig 6:**
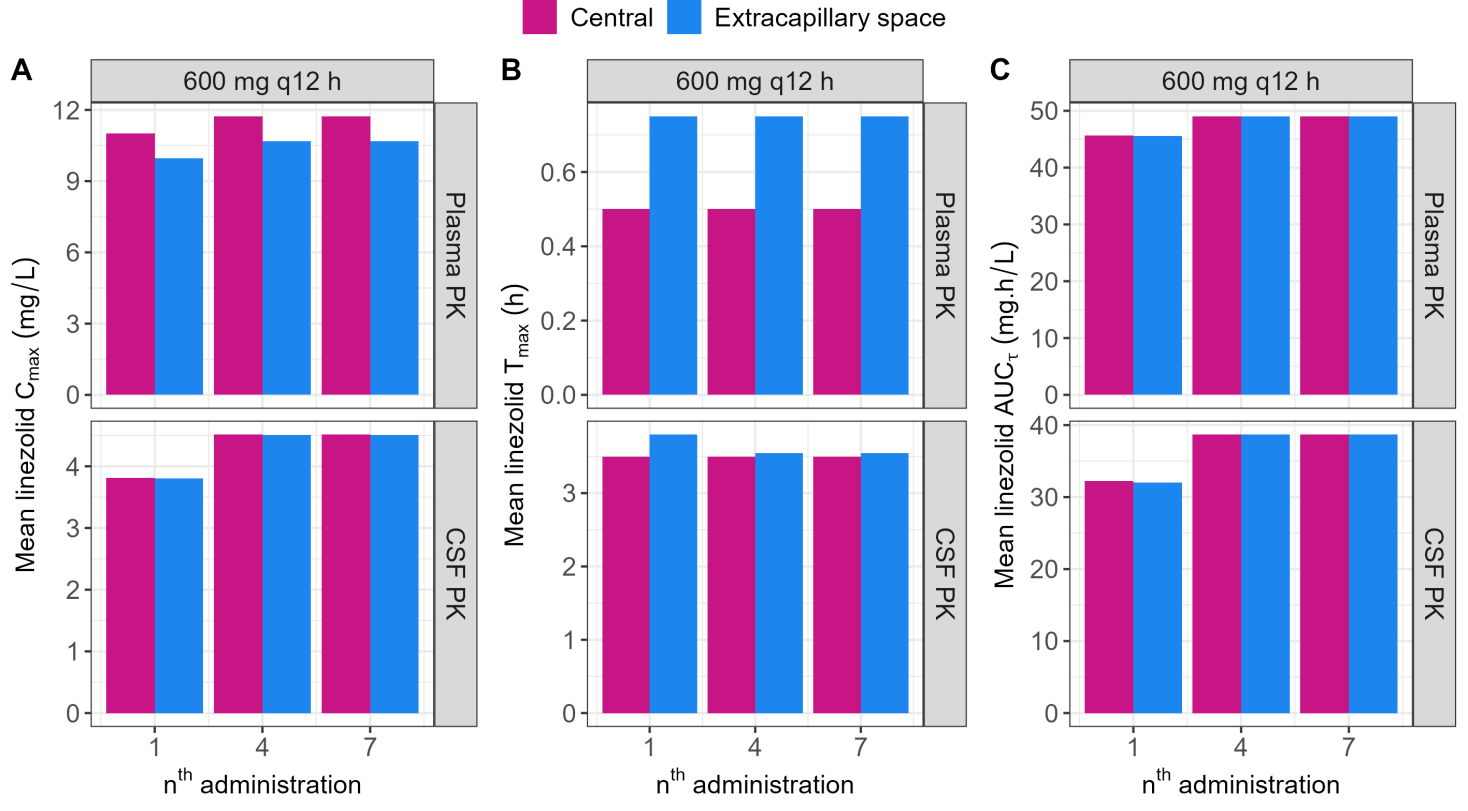
Linezolid PK parameters computed from observations in the central reservoir and in the cartridge ECS from HFIM experiments reproducing plasma PK (top panels) and CSF PK (bottom panels) after administration of 600 mg q12 h (replicate number = 2). (**A**) Mean observed maximal concentrations (*C*_max_) of linezolid in the central reservoir (pink) and cartridge ECS (blue) after the first, third, fifth, and seventh administration. (**B**) Mean observed time to reach *C*_max_ (*T*_max_) of linezolid in the central reservoir (pink) and cartridge ECS (blue) after the first, third, fifth, and seventh administration. (**C**) Mean observed area under the curve over the dosing interval (AUC_τ_) of linezolid in the central reservoir (pink) and cartridge ECS (blue) after the first, third, fifth, and seventh administration.

The tri-compartmental model estimated a diffusion flow rate (*Q*_diffusion_) at 6.45 mL/min (0.387 L/h). Diffusion from the ICS to the ECS was not instantaneous, resulting in *T*_max_ in the cartridge ECS being delayed by 0.250 h when compared with the central reservoir concentrations when reproducing linezolid plasma concentrations, whereas no difference was found when reproducing CSF concentrations (Fig. 6B).

When reproducing plasma concentrations, this delay prevented complete diffusion of linezolid to the cartridge ECS, resulting in an ~10% lower observed *C*_max_ in the cartridge ECS when compared with the central reservoir concentrations, whereas no difference was found when reproducing CSF concentrations (Fig. 6A).

However, there was no impact on antibiotic exposure since the AUC_τ_ was the same between central reservoir and cartridge ECS for all simulated concentrations (Fig. 6C).

Comparable results were observed for 900 mg q12 h and 900 mg q8 h dosing regimens (Fig. S1 and S2).

### Hollow-fiber setup can be used to reproduce linezolid plasma PK

**Fig 7:**
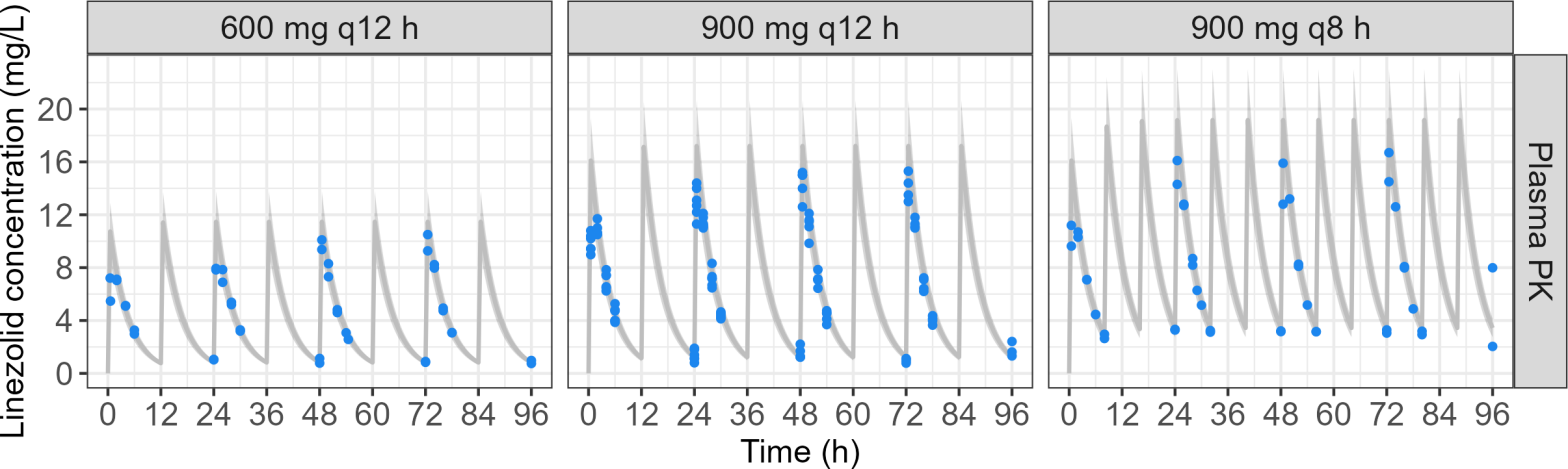
Target linezolid plasma concentrations from the linezolid clinical population PK model (13) and observed linezolid concentrations in the cartridge ECS after infusion of 600 mg q12 h, 900 mg q12 h, and 900 mg q8 h (replicate number = 2–6). The gray line corresponds to target linezolid concentrations. The gray area corresponds to a 20% bias from target linezolid concentrations. Points correspond to linezolid concentrations observed in the cartridge ECS of the HFIM.

Target linezolid plasma concentrations from linezolid clinical population PK model (13) and observed linezolid concentrations in the cartridge ECS after an administration of 600 mg q12 h, 900 mg q12 h, and 900 mg q8 h are shown in Fig. 7. The overall bias of the observed vs predicted concentrations was −3.1% ± 14.6% for 600 mg q12 h, −7.8% ± 20.8% for 900 mg q12 h, and −6.9% ± 23.7% for 900 mg q8 h.

Target and observed linezolid plasma PK parameters are compared in Table 3. *C*_max,1_, *C*_max,ss_, *t*_1/2_, AUC_τ, 1_, and AUC_τ, ss_ showed a bias lower than 10%. *T*_max,1_ and *T*_max,ss_ absolute bias was 0.25 h (0.5 vs 0.75 h), which we deemed acceptable.

**TABLE 3:** Target linezolid plasma PK parameters vs mean observed cartridge ECS linezolid PK parameters

	Plasma								
	600 mg q12 h			900 mg q12 h			900 mg q8 h		
	Target	ECS	Bias (%)	Target	ECS	Bias (%)	Target	ECS	Bias (%)
*C*_max, 1_ (mg/L)	10.7	9.97	−7.08	16.1	14.8	−7.82	16.1	14.6	−9.60
*T*_max, 1_ (h)	0.500	0.750	0.250^*a*^	0.500	0.750	0.250^*a*^	0.500	0.750	0.250^*a*^
AUC _τ, 1_ (mgxh/L)	46.1	45.6	−1.24	69.2	67.8	−2.02	61.6	58.9	−4.29
*C*_max, ss_ (mg/L)	11.5	10.7	−6.89	17.2	15.9	−7.63	19.2	17.4	−9.11
*T*_max, ss_ (h)	24.5	24.8	0.250^*a*^	24.5	24.8	0.250^*a*^	24.5	24.8	0.250^*a*^
AUC _τ, ss_ (mgxh/L)	49.5	49.0	−1.06	74.3	72.9	−1.84	74.3	71.5	−3.74
*C*_min,ss_ (mg/L)	0.821	0.834	1.66	1.23	1.24	0.856	3.42	3.39	−0.97
*t*_1/2_ (h)	3.01	3.03	0.511	3.01	3.03	0.511	3.01	3.03	0.511

^*a*^ For *T*_max_, absolute bias (in hours) was reported.

### Hollow-fiber setup can be used to reproduce linezolid CSF PK

**Fig 8:**
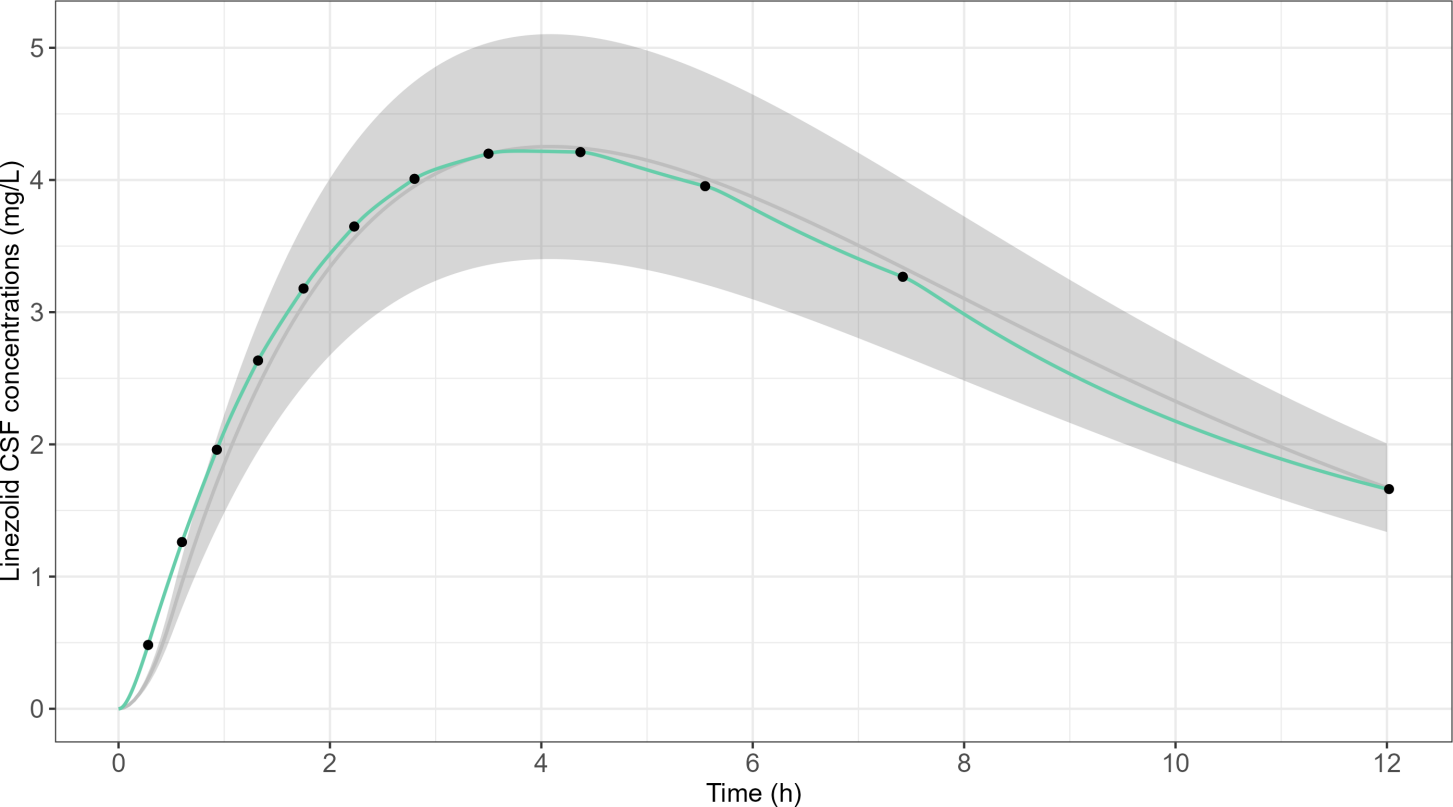
Target linezolid CSF concentrations from linezolid clinical population PK model (13) vs expected linezolid concentrations in the cartridge ECS for the first dose of 600 mg q12 h dosing regimen. The gray line corresponds to target linezolid concentrations. The gray area corresponds to a bias of 20% from the target linezolid concentrations. The green line corresponds to expected linezolid concentrations in the cartridge ECS with the infusion pump program shown in Table 4. Black points correspond to expected concentrations at the end of each infusion sub-interval.

The ambulatory infusion pump program to reproduce CSF concentrations after administration of 600 mg q12 h of linezolid is presented in Table 4. The corresponding expected PK profile after the first dose in the cartridge ECS is presented in Fig. 8.

**TABLE 4:** Ambulatory infusion pump program to reproduce CSF concentrations after administration of 600 mg q12 h of linezolid

Sub-interval number	Sub-interval duration (min)	Infusion pump flow rate (Flow_infusion,i_) (mL/h)	Amount infused (mg)
1	17	7.1	0.297
2	19	6.5	0.297
3	20	5.9	0.297
4	23	5.3	0.297
5	26	4.7	0.297
6	29	4.1	0.297
7	34	3.5	0.297
8	42	2.9	0.297
9	52	2.3	0.297
10	71	1.7	0.297
11	112	1.1	0.297
12	276	0.4	0.297

Overall, the computed infusion pump program provided a good approximation of the target CSF concentrations. Comparable results were observed for the 900 mg q12 h and 900 mg q8 h dosing regimens (Table S3; Fig. S3).

The bias and imprecision of the expected concentration profiles with the infusion program computed with the algorithm are presented in Table S4.

**Fig 9:**
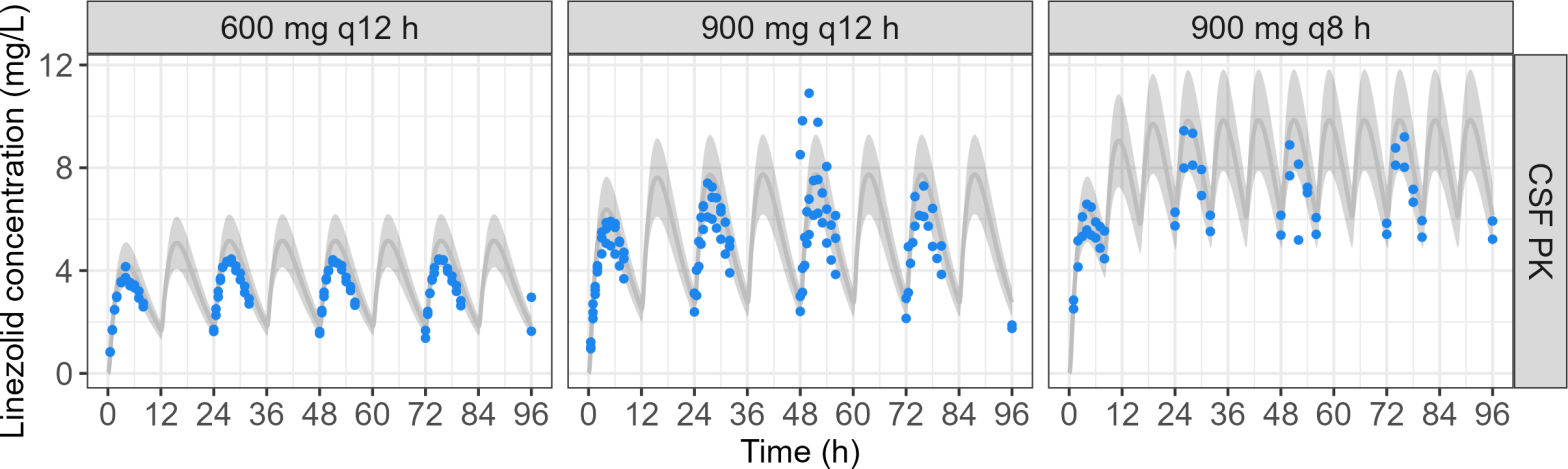
Target linezolid CSF concentrations from linezolid clinical population PK model (13) and observed linezolid concentrations in the cartridge ECS after infusion of 600 mg q12 h, 900 mg q12 h, and 900 mg q8 h (replicate number = 2–3). The gray line corresponds to target linezolid concentrations. The gray area corresponds to a 20% bias from target linezolid concentrations. Points correspond to linezolid concentrations observed in the cartridge ECS of the HFIM.

Target linezolid CSF concentrations from linezolid clinical population PK model (13) and observed linezolid concentrations in the cartridge ECS after an administration of 600 mg q12 h, 900 mg q12 h, and 900 mg q8 h are shown in Fig. 9. Despite being lower than expected, these concentrations were within the 20% bias range, and the overall bias of the observed vs predicted concentrations was −11.3% ± 10.8% for 600 mg q12 h, −5.0% ± 31.3% for 900 mg q12 h, and −7.5% ± 9.7% for 900 mg q8 h.

Target and observed linezolid CSF PK parameters are compared in Table 5. *C*_max,1_, *C*_max,ss_, *t*_1/2_, AUC_τ, 1_, and AUC_τ, ss_ showed a bias lower than 20%. *T*_max,1_ absolute bias was 0.3 h or less, which we deemed acceptable. *T*_max,ss_ was unbiased.

**TABLE 5:** Target linezolid CSF PK parameters vs observed cartridge ECS linezolid PK parameters

	CSF								
	600 mg q12 h			900 mg q12 h			900 mg q8 h		
	Target	ECS	Bias (%)	Target	ECS	Bias (%)	Target	ECS	Bias (%)
*C*_max, 1_ (mg/L)	4.25	3.80	−10.6	6.38	5.60	−12.1	6.38	6.36	−0.276
*T*_max, 1_ (h)	4.10	3.80	−0.300^*a*^	4.10	3.80	−0.300^*a*^	4.10	4.00	−0.100^*a*^
AUC _τ, 1_ (mgxh/L)	35.9	32.0	−10.9	53.9	47.2	−12.3	39.7	40.3	−1.55
*C*_max, ss_ (mg/L)	5.16	4.50	−12.6	7.73	6.64	−14.1	9.83	9.67	−1.68
*T*_max, ss_ (h)	27.6	27.6	0.000^*a*^	27.6	27.6	0.000^*a*^	27.0	26.9	−0.0500^*a*^
AUC _τ, ss_ (mgxh/L)	44.7	38.7	−13.4	67.0	57.1	−14.8	66.9	67.1	0.403
*C*_min,ss_ (mg/L)	1.82	1.59	−12.7	2.73	2.35	−14.0	6.05	5.97	−1.41
*t*_1/2_ (h)	3.01	3.03	0.511	3.01	3.03	0.511	3.65	3.66	0.268

^*a*^ For *T*_max_, absolute bias (in hours) was reported.

## DISCUSSION

Most HFIM studies were found in the literature to assay drug concentrations in the central reservoir rather than in the ECS (4, 5, 7–9, 28–32). However, since bacteria are trapped within the ECS, it represents the true site of action; thus, concentrations in the ECS are the most relevant to PK/PD studies. The reason why most studies do not assay drugs in the ECS is that it is usually assumed that concentrations in the ECS are in very rapid equilibrium with the central reservoir concentrations.

Some studies challenged this assumption by measuring concentrations in both the central reservoir and ECS and reported that a delay of 15 (6) and 20 min (18) was necessary to reach equilibrium between central reservoir and the ECS of the cartridge after short infusions of 30 min or 1 h. However, in those studies, they did not evaluate the impact of dosing regimen and half-life on the observed delay, which we were able to do here by comparing the reproduction of plasma and CSF concentrations in hollow fiber.

In line with those previous reports, we also observed a delay of 15 min (0.25 h) in reaching equilibrium between the central reservoir and ECS when we reproduced 30-min short infusions of linezolid (Fig. 6B). However, when reproducing CSF PK profiles, no such delay was observed (Fig. 6B). Our hypothesis is that this delay is proportional to the rate of change in central reservoir concentrations, where the higher the infusion rate and the lower the half-life, the longer the time to equilibrium will be. This delay not only has an impact on the time necessary to reach maximal concentration (*T*_max_) in the ECS but also on the maximal concentration itself (*C*_max_). Indeed, while the drug concentrations equilibrate between the central reservoir and the ECS, the drug is also eliminated from the central reservoir, reducing the overall amount of drug that reaches the ECS. In our study, the impact on *C*_max_ was limited (<10%, Table 3). However, if the delay is truly proportional to infusion rate and half-life, there will be future cases where the impact of *C*_max_ will be significant. In such cases, the parameterization of the HFIM could be adjusted in order to take into account this equilibration delay. In order to do so, one would have to first estimate the rate of diffusion from the ICS to the ECS (*Q*_diffusion_) and then perform numerical optimization to find the best HFIM parameters to reproduce the desired concentration-time curve. Our results further reinforce the need to measure ECS concentrations when performing HFIM experiments when one wants to study the PK/PD of an anti-infective drug. Furthermore, it is especially important for antibiotics whose physicochemical properties render susceptible to sticking to hollow -fibers (26) or to bacteria-mediated degradation (e.g., β-lactamase-producing strains) (33).

CSF concentrations reproduced in the HFIM were systematically lower than expected (Fig. 9). This can be explained by the algorithm used, which allows only a fraction of the target dose to be administered at the end of the last sub-interval (*f*_dose_), systematically leading to lower than expected concentrations. This fraction was lower for the 900 mg q8h regimen (*f*_dose_ ≈ 90%) compared to the q12h regimens (*f*_dose_ ≈ 97%), related to the technical limitations of our ambulatory pump, which restricted us to use 8 sub-intervals instead of 12 for other dosing regimens (Table S2). Despite this limitation, we were able to reproduce the targeted CSF concentrations with a bias of less than 20%. Another limitation of our validation is that when we computed the target PK parameters to reproduce linezolid CSF concentrations in the HFIM, we empirically adjusted the fitted *k*_*e*_ and *k*_*a*_ values to yield a better match between the expected concentrations and the target concentrations. However, even though we made empirical adjustments, bias of the observed PK parameters remained lower than 20% for all tested dosing regimens (*n* = 3), target PK (*n* = 2, plasma and CSF per dosing regimen), and all replicates (*n* ≥2 for each setup) (Tables 3 and 5).

We developed and validated a new HFIM setup that enables *in vitro* reproduction of mono-compartmental PK with an absorption phase. This setup was applied to successfully reproduce site-of-action PK of linezolid but could also be used to reproduce plasma PK after oral administration. An important strength of our setup is that it does not come with an overly complex modification to the material setup of the experiment, adding only a programmable infusion pump to the system (as opposed to adding a whole compartment and peristaltic pump).

This was made possible by the development of an algorithm that computes infusion rates and durations that enable an approximation of the first-order absorption kinetics by a series of continuous infusions. Since manual application of the algorithm would be tedious, we included it in an open-source web application designed to help design experimental protocols for HFIM (25). Thus, our setup and algorithm are easy to translate to any other HFIM experiment. This is not the first application that aims to help researchers to perform hollow-fiber experiments (16). Compared to the work by Klopprogge et al., our app proposes several improvements including: the ability to apply the currently proposed algorithm for first-order absorption kinetics; the ability to download all plots, tables, and simulations; and the openness of the code, which is distributed under an open license and available at: https://github.com/INSERM-U1070-PHAR2/HF-App. It should be noted that the app from Klopprogge et al. proposes two functionalities that ours does not: they propose the option to simplify the two-compartment system by using a zero-order infusion into the central reservoir for the drug with the longer half-life and have an option to start the experiment from steady state. We acknowledge the pertinence of these options and will include them in an updated version of the app.

## Data Availability

A tutorial for use of the shiny app is provided in Text S3. The data used in this study are available at https://doi.org/10.57745/NRXPOP. R code for the shiny application can be found on GitHub: https://github.com/INSERM-U1070-PHAR2/HF-App. It has also been archived on the Software Heritage website: https://archive.softwareheritage.org/swh:1:dir:f8177c3793324b996d14d7a1bf0490a4b2a69554.

## SUPPLEMENTAL MATERIAL

Code S1 spectrum.00051-25-s0001.txt R code for the shiny application.

Supplemental material spectrum.00051-25-s0002.docx Supplemental tables, text, and tutorial of the HF application.

ASM does not own the copyrights to Supplemental Material that may be linked to, or accessed through, an article. The authors have granted ASM a non-exclusive, world-wide license to publish the Supplemental Material files. Please contact the corresponding author directly for reuse.
