# Supplementary material for: A freely accessible, adaptable hollow-fiber setup to reproduce first-order absorption: illustration with linezolid cerebrospinal fluid pharmacokinetic data
Source: Microbiol Spectr. 2025 May 15;13(6):e00051-25. doi: 10.1128/spectrum.00051-25 (PMC12131858; doi:10.1128/spectrum.00051-25)
Supplement: Supplemental material — Supplemental tables, text, and tutorial of the HF application. [file spectrum.00051-25-s0002.docx]

# Supplementary data

## Table S1: Experimental parameters used in HFIM to simulate plasma concentrations

|  | **Parameter** | **Parameter description** | **600 mg q12 h** | **900 mg q12 h** | **900 mg q8 h** |
| --- | --- | --- | --- | --- | --- |
| Set in the application | V_Central_ (L) | Volume in the central reservoir | 0.300 | 0.300 | 0.300 |
|  | Q_Cartridge_ (L/h) | Pump flow rate from the central reservoir to the cartridge | 3.60 | 3.60 | 3.60 |
|  | V_Cartridge_ (L) | Volume of the cartridge | 0.0600 | 0.0600 | 0.0600 |
|  | t_infusion_ (h) | Infusion duration | 0.500 | 0.500 | 0.500 |
|  | V_infusion_ (mL) | Volume of infusion | 5.00 | 5.00 | 5.00 |
|  | τ (h) | Dosing interval | 12 | 12 | 8 |
|  | n_doses_ | Total number of doses | 8 | 8 | 12 |
|  | Exp_duration_ (h) | Experiment duration | 96 | 96 | 96 |
|  | C_max,1_ (mg/L) | Maximal concentration after the first dose | 10.7 | 16.1 | 16.1 |
|  | t_1/2_ (h) | Terminal half-life | 3.01 | 3.01 | 3.01 |
| Computed by the application | CL_elim_ (L/h) | Pump flow rate from the central reservoir to the waste | 0.0828 | 0.0828 | 0.0828 |
|  | V_diluent_ (L) | Volume of diluent | 7.95 | 7.95 | 7.95 |
|  | C_infusion_ (mg/L) | Infusion solution concentration | 838 | 1220 | 1230 |
| Performed | Replicates | Number of replicates | 2 | 6 | 2 |

## Table S2: Experimental parameters used in HFIM to simulate CSF concentrations

|  | **Parameter** | **Parameter description** | **600 mg q12 h** | **900 mg q12 h** | **900 mg q8 h** |
| --- | --- | --- | --- | --- | --- |
| Set in the application | V_Central_ (L) | Volume in the central reservoir | 0.300 | 0.300 | 0.300 |
|  | Q_Cartridge_ (L/h) | Pump flow rate from the central reservoir to the cartridge | 3.60 | 3.60 | 3.60 |
|  | V_Cartridge_ (L) | Volume of the cartridge | 0.0600 | 0.0600 | 0.0600 |
|  | t_1/2_ (h) | Terminal half-life | 3.01 | 3.01 | 3.65 |
|  | F | Bioavailability | 1 | 1 | 1 |
|  | k_a_ (h^-1^) | Absorption rate constant | 0.300 | 0.300 | 0.300 |
|  | C_max, 1_ (mg/L) | Maximal concentration after the first dose | 4.25 | 6.38 | 6.38 |
|  | Exp_duration_ (h) | Experiment duration | 96 | 96 | 96 |
|  | $n$ | Number of sub-intervals | 12 | 12 | 8 |
|  | t_n_ (h) | The end time of the last sub-interval | 12 | 12 | 8 |
|  | n_doses_ | Total number of doses | 8 | 8 | 12 |
|  | V_infusion_ (mL) | Volume of infusion | 2.00 | 2.00 | 2.00 |
| Computed by the application | t_max,1_ (h) | Time to reach C_max,1_ | 3.80 | 3.80 | 4.15 |
|  | Dose (mg) | Dose administered at the end of the last sub-interval | 3.66 | 5.50 | 5.06 |
|  | f_dose_ | Fraction of target dose administered at the end of the last sub-interval | 0.973 | 0.973 | 0.909 |
|  | CL_elim_ (L/h) | Pump flow rate from the central reservoir to the waste | 0.0828 | 0.0828 | 0.0684 |
|  | V_diluent_ (L) | Volume of diluent | 7.95 | 7.95 | 6.57 |
|  | C_infusion_ (mg/L) | Infusion solution concentration | 148 | 222 | 288 |
| Performed | Replicates | Number of replicates | 2 | 3 | 2 |

## Table S3: Ambulatory infusion pump program to reproduce CSF concentrations after administration of 900 mg q12 h and 900 mg q 8 h of linezolid

| **900 mg q12 h** | | | | **900 mg q8 h** | | | |
| --- | --- | --- | --- | --- | --- | --- | --- |
| Sub-interval number | Sub-interval duration (min) | Infusion pump flow rate (Flow_infusion,i_) (mL/h) | Amount infused (mg) | Sub-interval number | Sub-interval duration (min) | Infusion pump flow rate (Flow_infusion,i_)  (mL/h) | Amount infused (mg) |
| 1 | 17 | 7.1 | 0.446 | 1 | 24 | 5.0 | 0.575 |
| 2 | 19 | 6.5 | 0.446 | 2 | 27 | 4.4 | 0.575 |
| 3 | 20 | 5.9 | 0.446 | 3 | 32 | 3.8 | 0.575 |
| 4 | 23 | 5.3 | 0.446 | 4 | 38 | 3.2 | 0.575 |
| 5 | 26 | 4.7 | 0.446 | 5 | 47 | 2.6 | 0.575 |
| 6 | 29 | 4.1 | 0.446 | 6 | 61 | 2.0 | 0.575 |
| 7 | 34 | 3.5 | 0.446 | 7 | 88 | 1.4 | 0.575 |
| 8 | 42 | 2.9 | 0.446 | 8 | 162 | 0.7 | 0.575 |
| 9 | 52 | 2.3 | 0.446 |  |  |  |  |
| 10 | 71 | 1.7 | 0.446 |  |  |  |  |
| 11 | 112 | 1.1 | 0.446 |  |  |  |  |
| 12 | 276 | 0.4 | 0.446 |  |  |  |  |

## Text S1: Detailed explanation of our mathematical algorithm

**Objective**

Find an experimental setup to be able to simulate first-order absorption and elimination within a hollow-fiber experiment.

**Setup**

Use the standard two-compartment hollow-fiber system (central reservoir + hollow fiber cartridge) with a variable flow infusion to simulate the drug absorption

**Challenges**

The challenge with the aforementioned setup is to find a procedure to compute the infusion parameters (*i.e.* concentration, volume and flow) with a focus on how often and how to change the infusion flow over time.

**Theoretical solution**

An analytical formula giving the infusion flow rate ($I\left( t \right)$) necessary to maintain a constant absorption rate parameter ($k_{a}\left( t \right)$) exists. It can be derived as follows:

$$\begin{matrix} k_{a}\left( t \right) & =\frac{I\left( t \right)}{V\left( t \right)} \\ k_{a}\left( t \right) & =\frac{I\left( t \right)}{V_{0}-I\left( t \right)\times t} \\ k_{a}\left( t \right) & =\frac{1}{\frac{V_{0}}{I\left( t \right)}-t} \\ \frac{1}{k_{a}\left( t \right)} & =\frac{V_{0}}{I\left( t \right)}-t \\ \frac{1}{k_{a}\left( t \right)}+t & =\frac{V_{0}}{I\left( t \right)} \\ I\left( t \right) & =\frac{V_{0}}{\frac{1}{k_{a}\left( t \right)}+t} \boldsymbol{(1)} \end{matrix}$$

Where: $t$ is the time post beginning of the infusion. $V\left( t \right)$ is the volume of drug solution remaining in the infusion bag at time $t$. $V_{0}$ is the initial volume in the infusion bag.

This equation is solvable for a known constant $k_{a}\left( t \right)$ and $V_{0}$*_._*

However, this solution is not applicable in our case because, we do not have access to a pump that allows for programming of a continuously varying infusion rate thus cannot apply this formula directly and instead need to approximate it by multiple discrete infusion rates changing at discrete times.

**Practical solution**

Let $n$ be the number of discrete infusions rates that we will use in the experiment. Let $t_{i}$ be the end time of sub-interval $i$ where $t\in[t_{i-1},t_{i}[$ and $t_{0}=0$ and $A_{i}$ be the amount of drug remaining to be administered for each $t_{i}$.

We need to define the duration of the dosing sub-intervals. We can define them by splitting the total dosing duration into $n$ sub*-*intervals in such a way that the amount of drug infused to the central reservoir during each sub-interval is equal. Mathematically:

$$\begin{matrix} A_{i} & =Dose-i\times\frac{Dose}{n} \boldsymbol{(3)} \\ Dose\times e^{-ka\times t_{i}} & =Dose-i\times\frac{Dose}{n} \\ Dose\times e^{-ka\times t_{i}} & =Dose\times\left( 1-\frac{i}{n} \right) \\ e^{-ka\times t_{i}} & =1-\frac{i}{n} \\ -ka\times t_{i} & =ln\left( 1-\frac{i}{n} \right) \\ t_{i} & =-\frac{ln\left( 1-\frac{i}{n} \right)}{k_{a}} \boldsymbol{(4)} \end{matrix}$$

However, in this setup when $i=n$, $t_{i}=+\infty$ which is longer than any sane experimentalist is willing to wait.

There are two ways to solve this issue. We ask the experimentalist to either define a fraction of the total dose that they want to be administered at the end of the last sub-interval, or specify the time at which the last sub-interval should end, in which case we will return the % of total dose administered by then. In this paper, since we are dealing with repeated doses with a predefined dosing interval, we chose the latter.

Let $f_{dose}$ be the fraction of total dose administered at the end of the last sub-interval ($t_{n}$). We can transform equation (**3**) to have $f_{dose}$ and $t_{n}$

$$\begin{matrix} A_{n} & =Dose-n\times f_{dose}\frac{Dose}{n} \\ A_{n} & =Dose-f_{dose}\times Dose \\ Dose\times e^{-ka\times t_{n}} & =Dose-f_{dose}\times Dose \\ Dose\times e^{-ka\times t_{n}} & =Dose\times\left( 1-f_{dose} \right) \\ e^{-ka\times t_{n}} & =1-f_{dose} \\ f_{dose} & =1-e^{-ka\times t_{n}} \boldsymbol{(5)} \end{matrix}$$

And in this case equation (**4**) has to be modified to take into account this forced end of last sub-interval while maintaining even sub-intervals with regards to amount transferred to central reservoir.

$$\begin{matrix} t_{i} & =-\frac{ln\left( 1-f_{dose}\frac{i}{n} \right)}{k_{a}} \end{matrix} \left( \boldsymbol{6} \right)$$

To put our solution into practice, we start by defining the time at which our last sub-interval will end ${(t}_{n})$, then we can use it in equation (**5**) to compute $f_{dose}$. Afterwards, we can use equation (**6**) to compute all $t_{i}$.

Then we can use the standard pharmacokinetic equation for first-order absorption to compute $A_{i}$ defined as:

$$A_{i}=Dose\times e^{-k_{a}\times t_{i}} \left( \boldsymbol{7} \right)$$

We can then simply compute the slope of the *A(t)* curve for each sub-interval and use this as our infusion rate, we will call it $S_{i}$:

$$S_{i}=\frac{A_{i-1}-A_{i}}{t_{i}-t_{i-1}} \left( \boldsymbol{8} \right)$$

This will allow $A_{i}$ to be equal to what it would have been with a first-order absorption process at the end of each sub-interval.

Bringing it all together we can write our algorithm in pseudocode as follows:

**Input:** number of sub-intervals $n$, desired maximal concentration after the first dose $C_{max,1}$, absorption rate constant $k_{a}$, terminal elimination half-life $t_{1/2}$, bioavailability $F$, end time of the last sub-interval $t_{n}$, central reservoir + hollow-fiber cartridge volume $V_{total}$.

**Output:** Table with $n$ rows with $t_{i}$, $A_{i}$, $S_{i}$, *Amount infused*, *Dose* and $f_{dose}$

1. $k_{e}=\frac{ln\left( 2 \right)}{t_{1/2}}$
2. $t_{max,1}=\frac{1}{k_{a}-k_{e}}ln\left( \frac{ka}{ke} \right)$
3. $Dose=C_{max,1}\times\left[ \frac{F\times k_{a}}{V_{total}\times\left( k_{a}-k_{e} \right)}\left( e^{-k_{e}\times t_{max,1}}-e^{-k_{a}\times t_{max,1}} \right) \right]^{-1}$
4. $f_{dose}=1-e^{-ka\times t_{n}}$

**for** $i$ in {1,2,…,n}

1. $t_{i}=-\frac{ln\left( 1-f_{dose}\frac{i}{n} \right)}{k_{a}}$
2. $A_{i}=Dose\times e^{-k_{a}\times t_{i}}$
3. $S_{i}=\frac{A_{i-1}-A_{i}}{t_{i}-t_{i-1}}$

**endfor**

**Example case**

$n=12$, $C_{max}=4.25mg/L$, $k_{a}=0.36h^{-1}$, $t_{1/2}=3.15h$,$V_{total}=0.360L$, $C=1000mg/L$, $F=1$, $t_{n}=12h$

| $t_{i}$ (h) | $A_{i}$ (mg) | $S_{i}$ (mg/h) | *Amount infused* (mg) |
| --- | --- | --- | --- |
| 0.00 | 3.32 | NA | NA |
| 0.24 | 3.04 | 1.14 | 0.27 |
| 0.50 | 2.77 | 1.05 | 0.27 |
| 0.79 | 2.50 | 0.95 | 0.27 |
| 1.11 | 2.23 | 0.85 | 0.27 |
| 1.47 | 1.95 | 0.75 | 0.27 |
| 1.89 | 1.68 | 0.65 | 0.27 |
| 2.38 | 1.41 | 0.55 | 0.27 |
| 2.98 | 1.14 | 0.46 | 0.27 |
| 3.74 | 0.86 | 0.36 | 0.27 |
| 4.80 | 0.59 | 0.26 | 0.27 |
| 6.52 | 0.32 | 0.16 | 0.27 |
| 12.00 | 0.04 | 0.05 | 0.27 |

$Dose$ = 3.3 mg $f_{dose}$ = 98.7%

## Text S2: Determination of fraction of fibers in the cartridge

Our goal is to compute the fraction of the total cartridge volume (F_ICS_) that is occupied by hollow-fibers (FX paed helixone dialyzer, Fresenius Medical Care, Bad Homburg, Germany).

From the cartridge documentation, we have:

| Fiber lumen diameter (cm) | Fiber wall thickness (cm) | Effective surface area (cm^2^) |
| --- | --- | --- |
| 0.0220 | 0.00350 | 2000 |

From direct measurements of the cartridge, we have:

| Cartridge length (cm) | Cartridge perimeter (cm) |
| --- | --- |
| 25.0 | 6.00 |

We will assume that the cartridge and the fibers are straight cylinders.

We can compute the volume of an individual fiber (V_fiber_):

$$V_{fiber}=\pi\times\frac{(lumen diamete{r+2\times wall thickness)}^{2}}{4}\times cartridge length$$

V_fiber_ = 0.017 cm^3^

We can also compute the surface area of 1 fiber (SA_fiber_):

$$SA_{fiber}=2 \times\pi\times\frac{(lumen diameter+2\times wall thickness)}{2}\times cartridge length$$

*Note that we omit the surface area of the horizontal cross section of the cylinder since it is unavailable for drug diffusion.*

SA_fiber_ = 2.28 cm^2^

In the cartridge documentation, we are provided with the effective surface area of the hollow fibers which is the sum of the surface area of all the hollow fibers. We can thus deduce the number of fibers in the cartridge n_fibers_:

$$n_{fibers}=\frac{Effective surface area}{SA_{fiber}}$$

n_fibers_ = 878

We can then compute the total volume occupied by the fibers (V_fibers,total_) by multiplying V_fiber_ by n_fibers_:

$$V_{fibers,total}=V_{fiber}\times n_{fibers}$$

V_fibers,total_ = 14.5 cm^3^

From the perimeter of the cartridge, we can compute the diameter of the cartridge (d_cartridge_):

$$d_{cartridge}=\frac{Cartridge perimeter}{\pi}$$

d_cartridge_ = 1.91 cm

We can then compute the theoretical volume of the cartridge:

$$V_{cartridge\_theoretical}=\pi\times\frac{d_{cartridge}^{2}}{4}\times cartridge length$$

V_cartridge_theoretical_ = 71.6 cm^3^

Finally, we can compute the fraction of cartridge volume occupied by the fibers:

$$F_{ICS}=\frac{V_{fibers,total}}{V_{cartridge\_theoretical}}$$

**F_ICS_ = 0.202**

## Text S3: Tutorial on how to use the HF-App to setup Linezolid 600q12 CSF concentrations

**Experimental setup:** Linezolid 600q12 in CSF

Parameters:

|  | **Parameter** | **Parameter description** | **600 mg q12 h** |
| --- | --- | --- | --- |
| Set in the application | V_Central_ (L) | Volume in the central reservoir | 0.300 |
|  | Q_Cartridge_ (L/h) | Pump flow rate from the central reservoir to the cartridge | 3.60 |
|  | V_Cartridge_ (L) | Volume of the cartridge | 0.0600 |
|  | t_1/2_ (h) | Terminal half-life | 3.01 |
|  | F | Bioavailability | 1 |
|  | k_a_ (h^-1^) | Absorption rate constant | 0.300 |
|  | C_max, 1_ (mg/L) | Maximal concentration after the first dose | 4.25 |
|  | Exp_duration_ (h) | Experiment duration | 96 |
|  | $n$ | Number of sub-intervals | 12 |
|  | t_n_ (h) | The end time of the last sub-interval | 12 |
|  | n_doses_ | Total number of doses | 8 |
|  | V_infusion_ (mL) | Volume of infusion | 2.00 |
| Computed by the application | t_max,1_ (h) | Time to reach C_max,1_ | 3.80 |
|  | Dose (mg) | Dose administered at the end of the last sub-interval | 3.66 |
|  | f_dose_ | Fraction of target dose administered at the end of the last sub-interval | 0.973 |
|  | CL_elim_ (L/h) | Pump flow rate from the central reservoir to the waste | 0.0828 |
|  | V_diluent_ (L) | Volume of diluent | 7.95 |
|  | C_infusion_ (mg/L) | Infusion solution concentration | 148 |

**Step by step procedure**

1. Go to : <https://varacli.shinyapps.io/hollow_fiber_app/> and wait for the app to load :


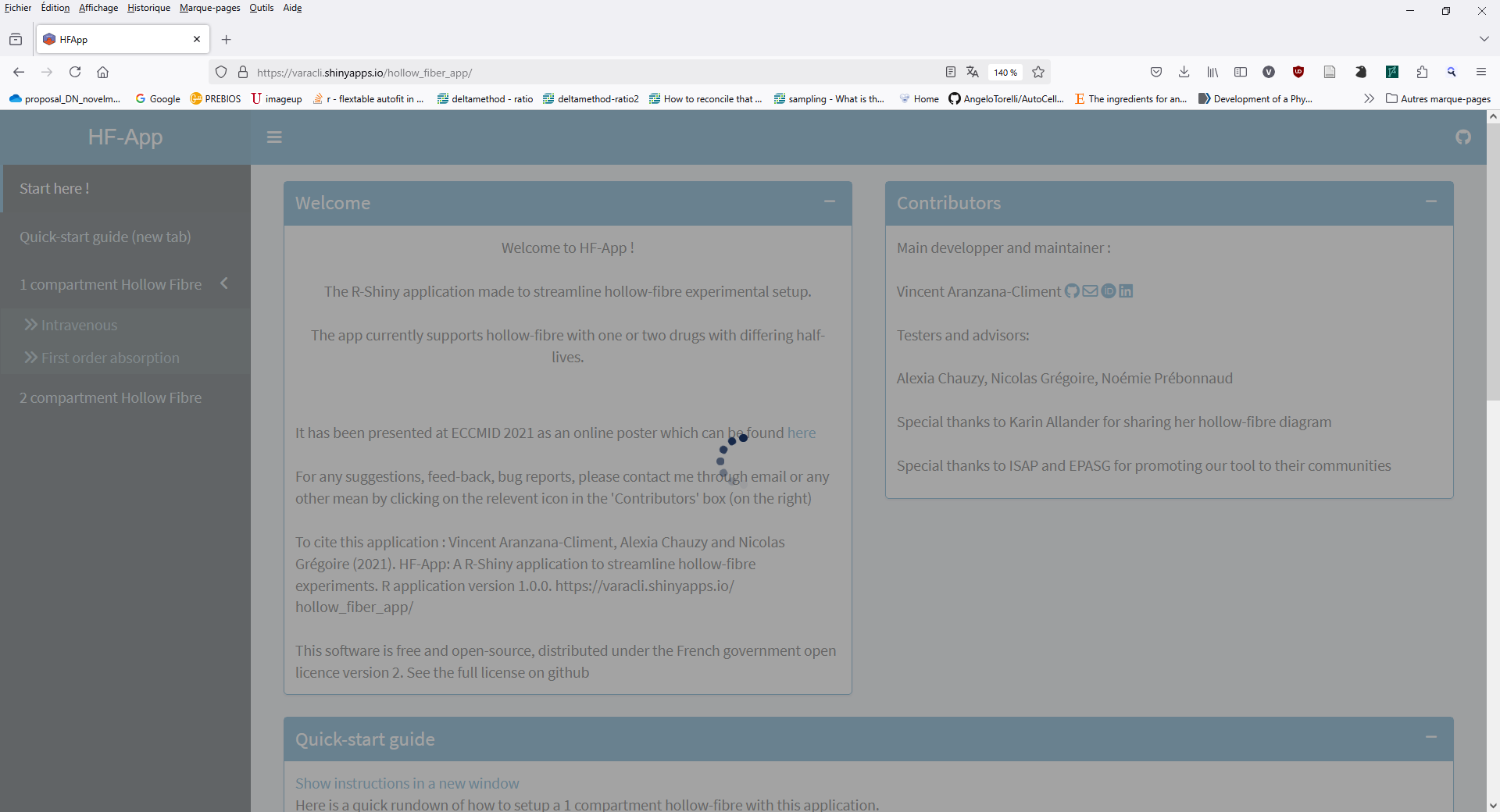


1. Click on « 1 compartment hollow fibre » → “First order absorption” on the sidebar


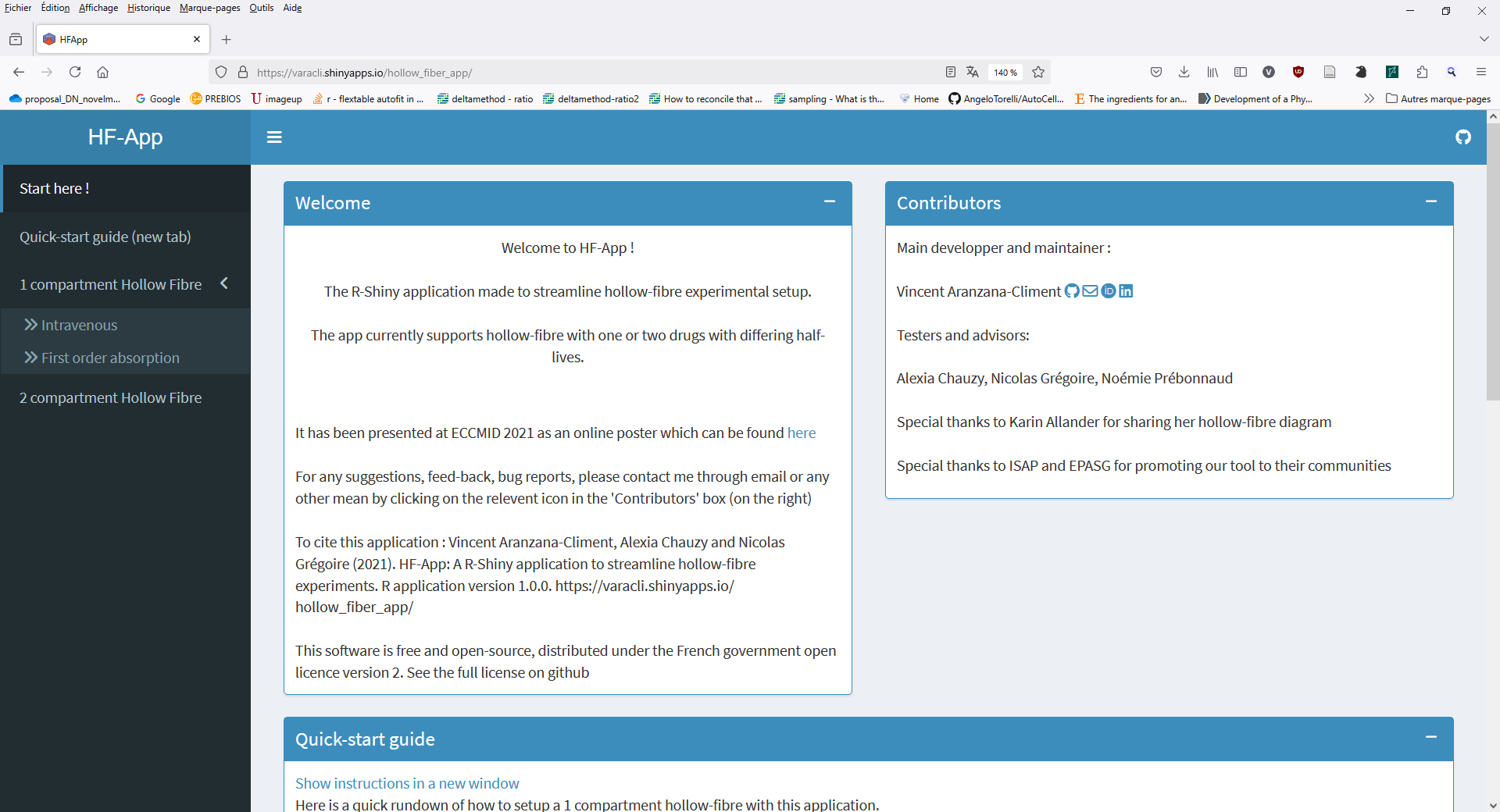


1. You are now on the “HF-Setup” tab


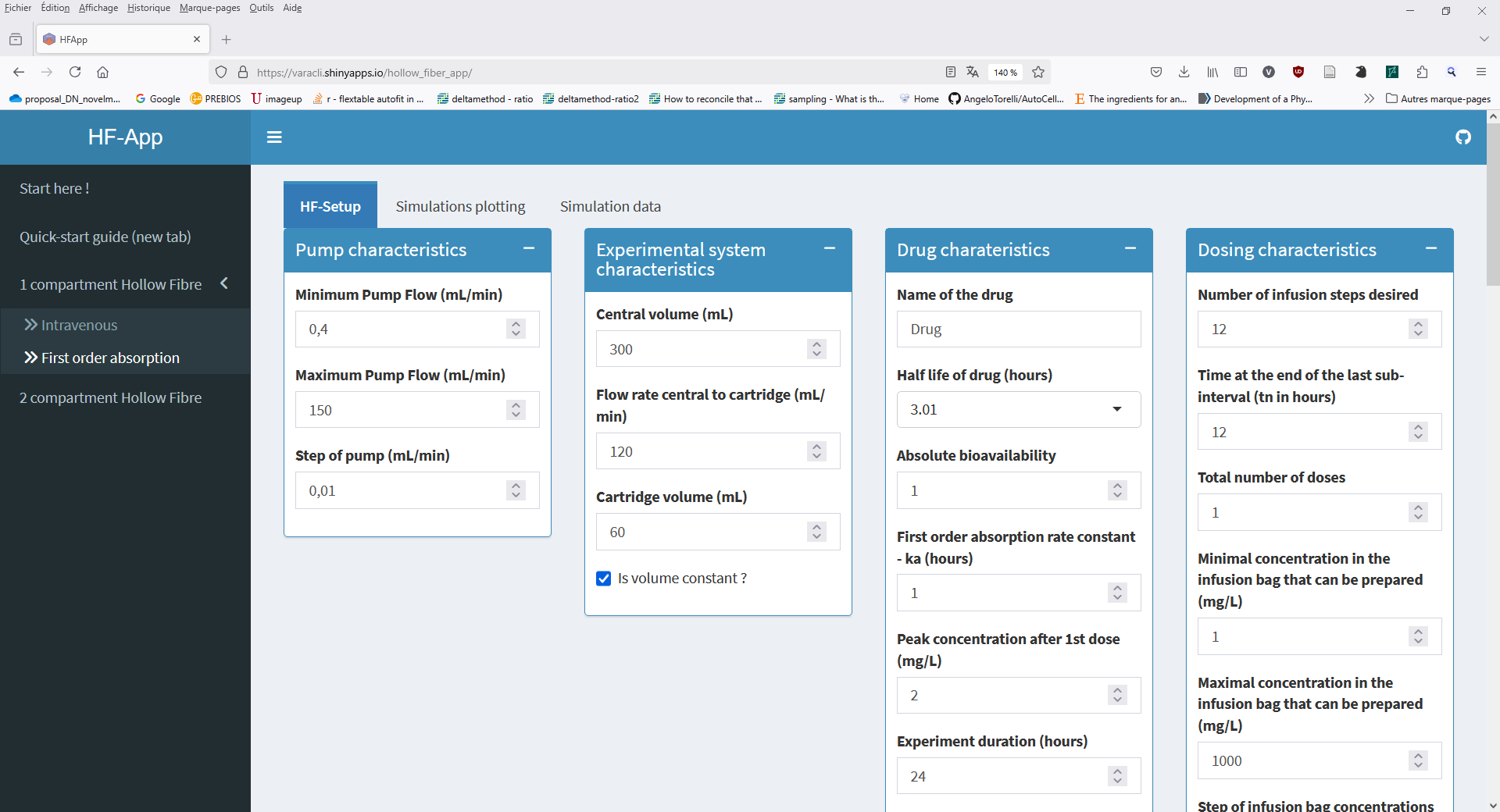


1. Input the technical specifications of your pump in order to limit which pump flows are available.


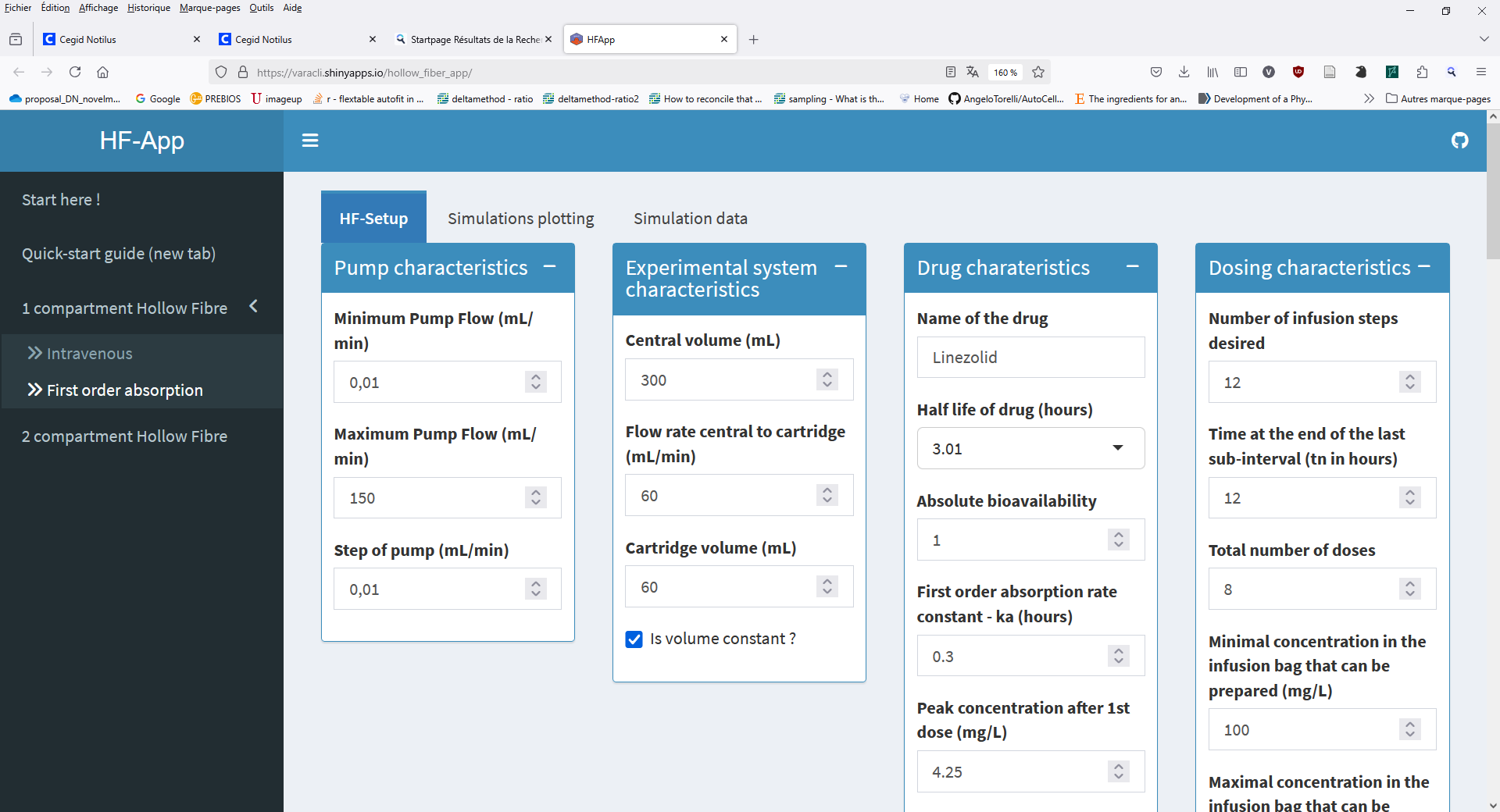


1. Input the Experimental system characteristics of your choice, in conjunction with pump characteristics it will be used to compute the half-lives that are available to you.


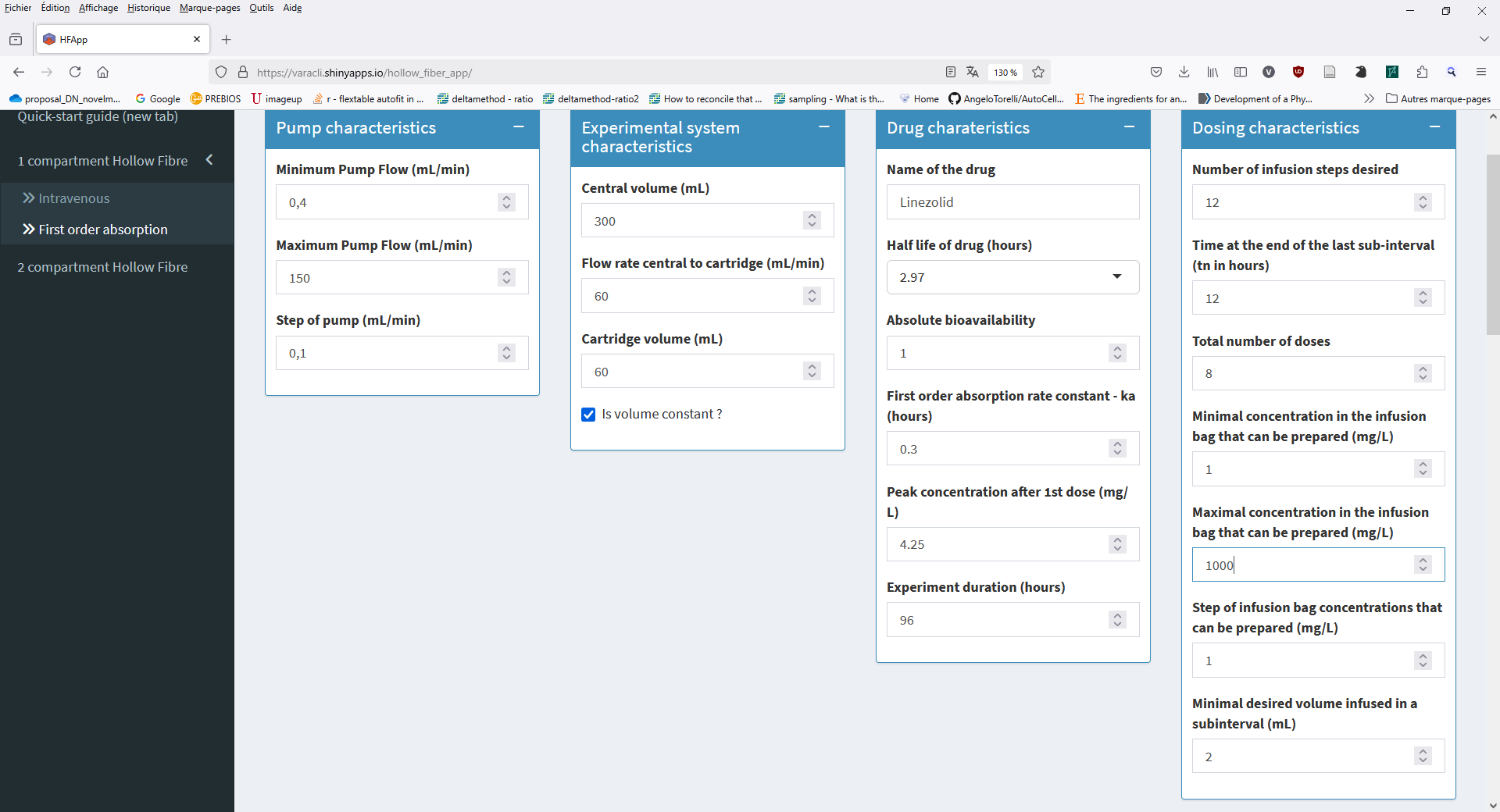


1. Input drug characteristics; please note that the half-life of the drug cannot be input manually, it has to be selected from the list since it depends on the pump characteristics and experimental volumes.


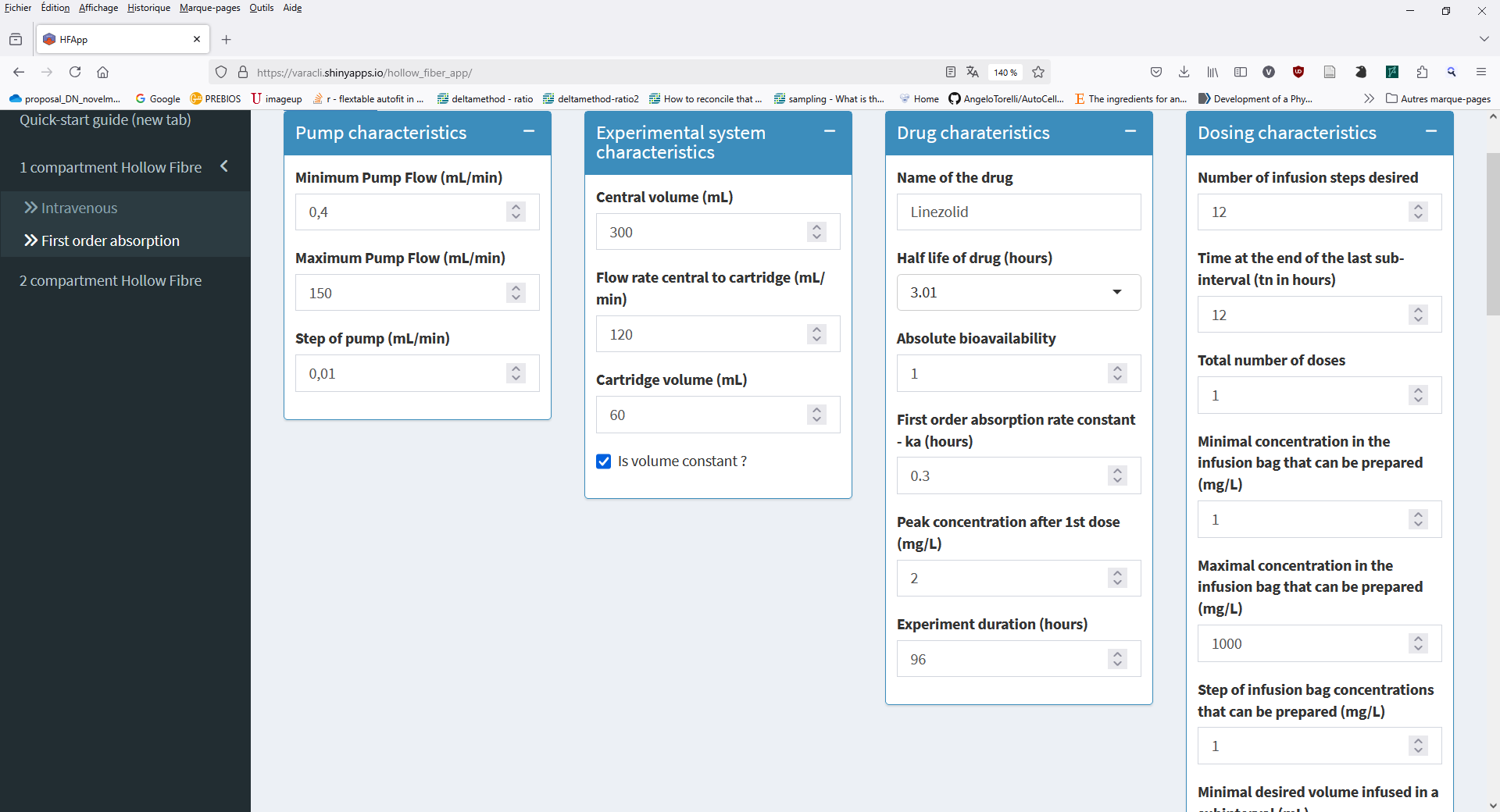


1. Input the desired dosing characteristics


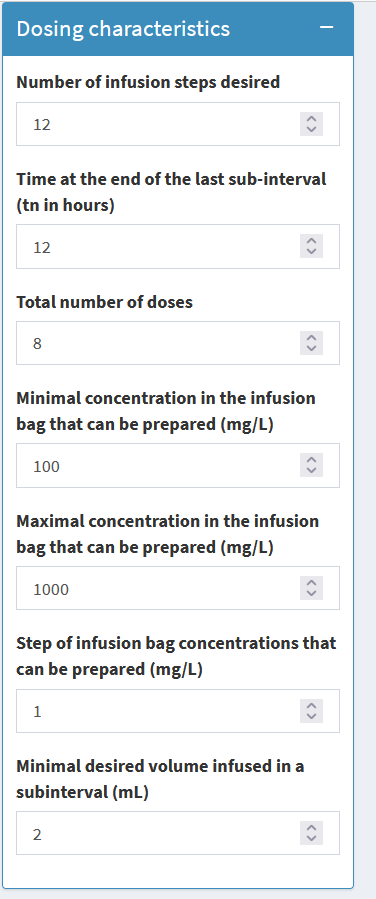


1. Click on the simulate HF button and wait for results.


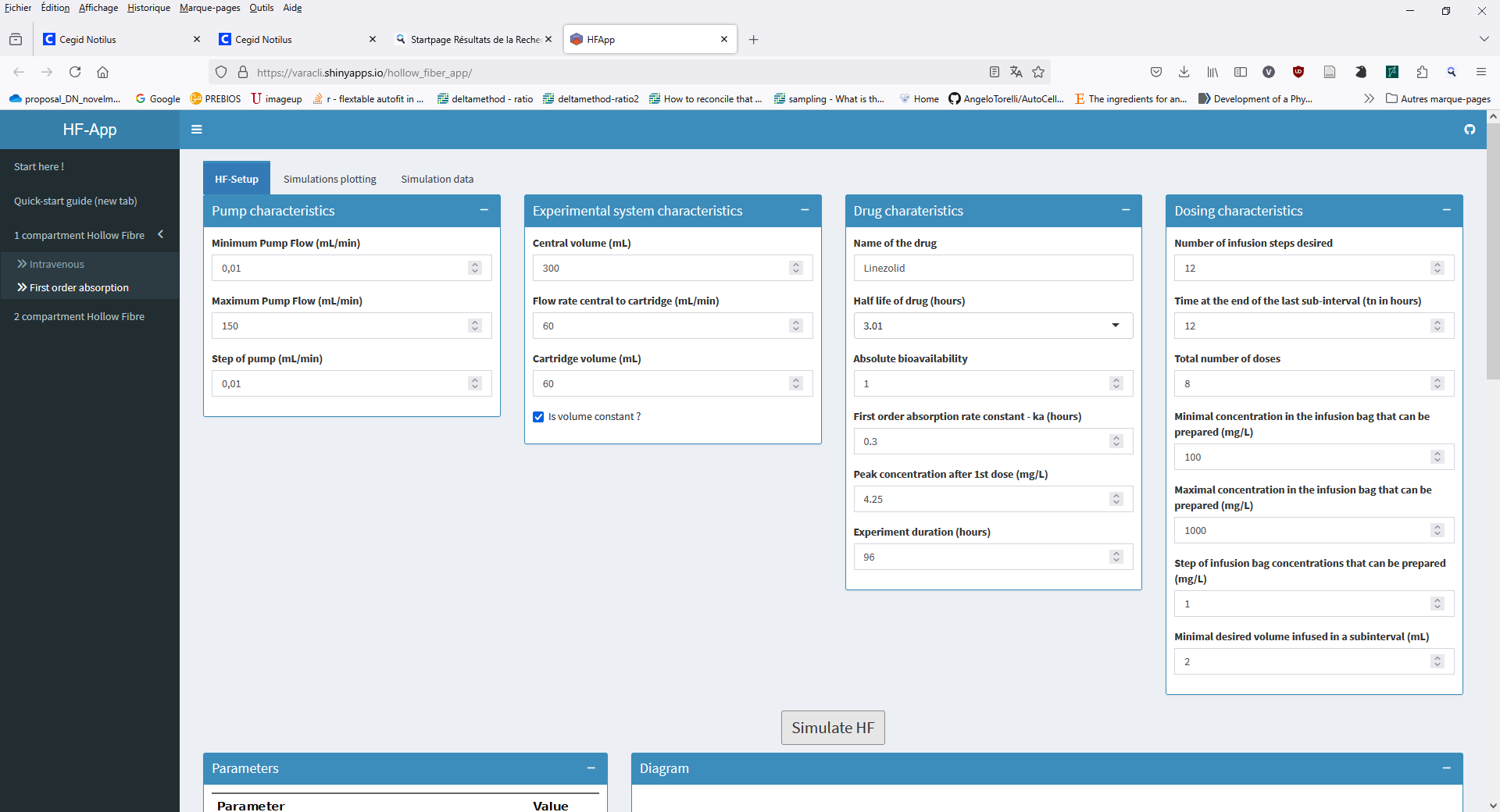


1. A table recapitulating all the experimental parameters and a diagram of the experiment are now available below the simulate button. You can download the table using the button at the bottom left, the diagram can be downloaded using the controls at the top right.


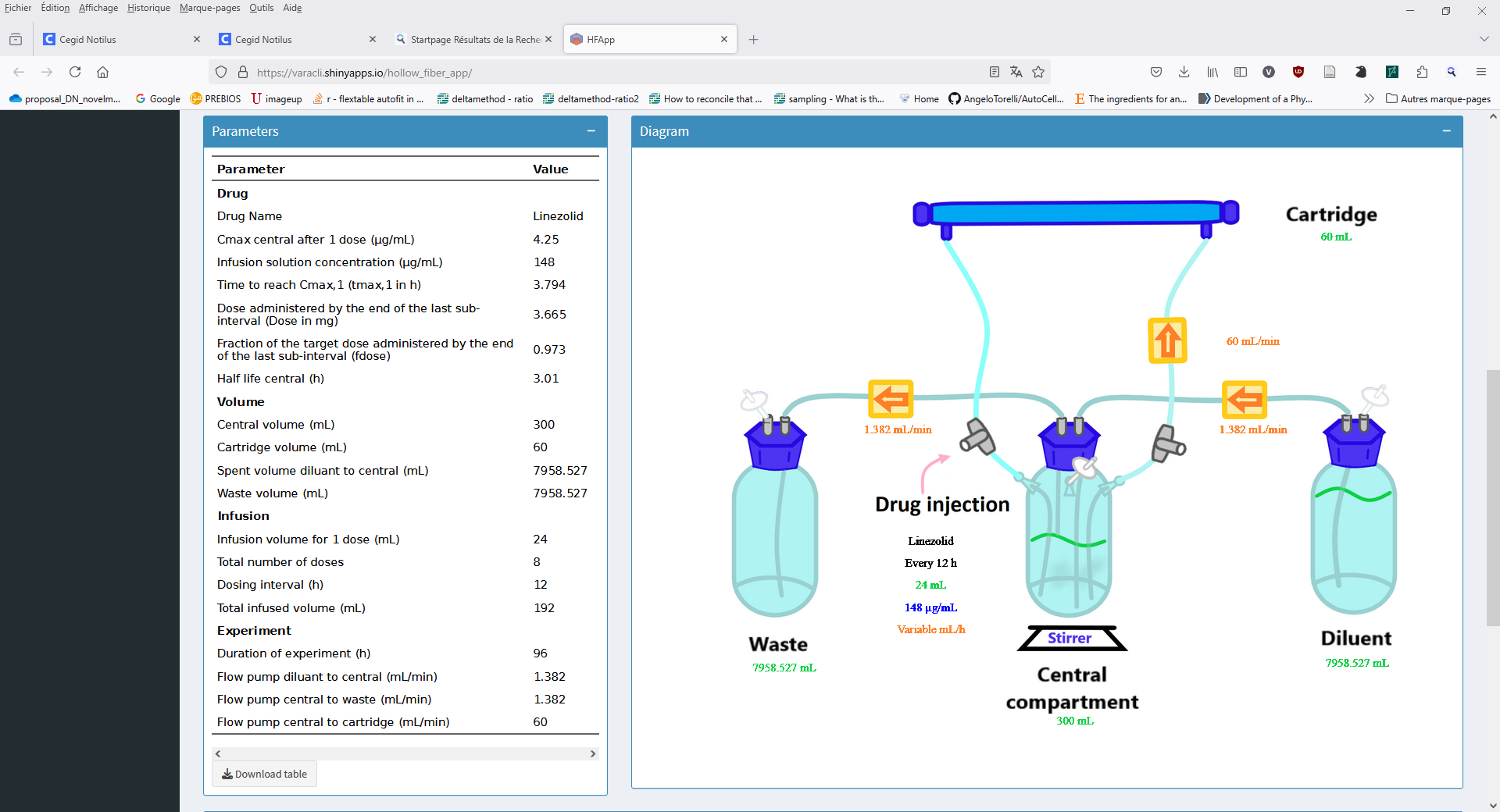


1. A table recapitulating the infusion program is shown at the bottom of the page with the option to download it at the bottom left.


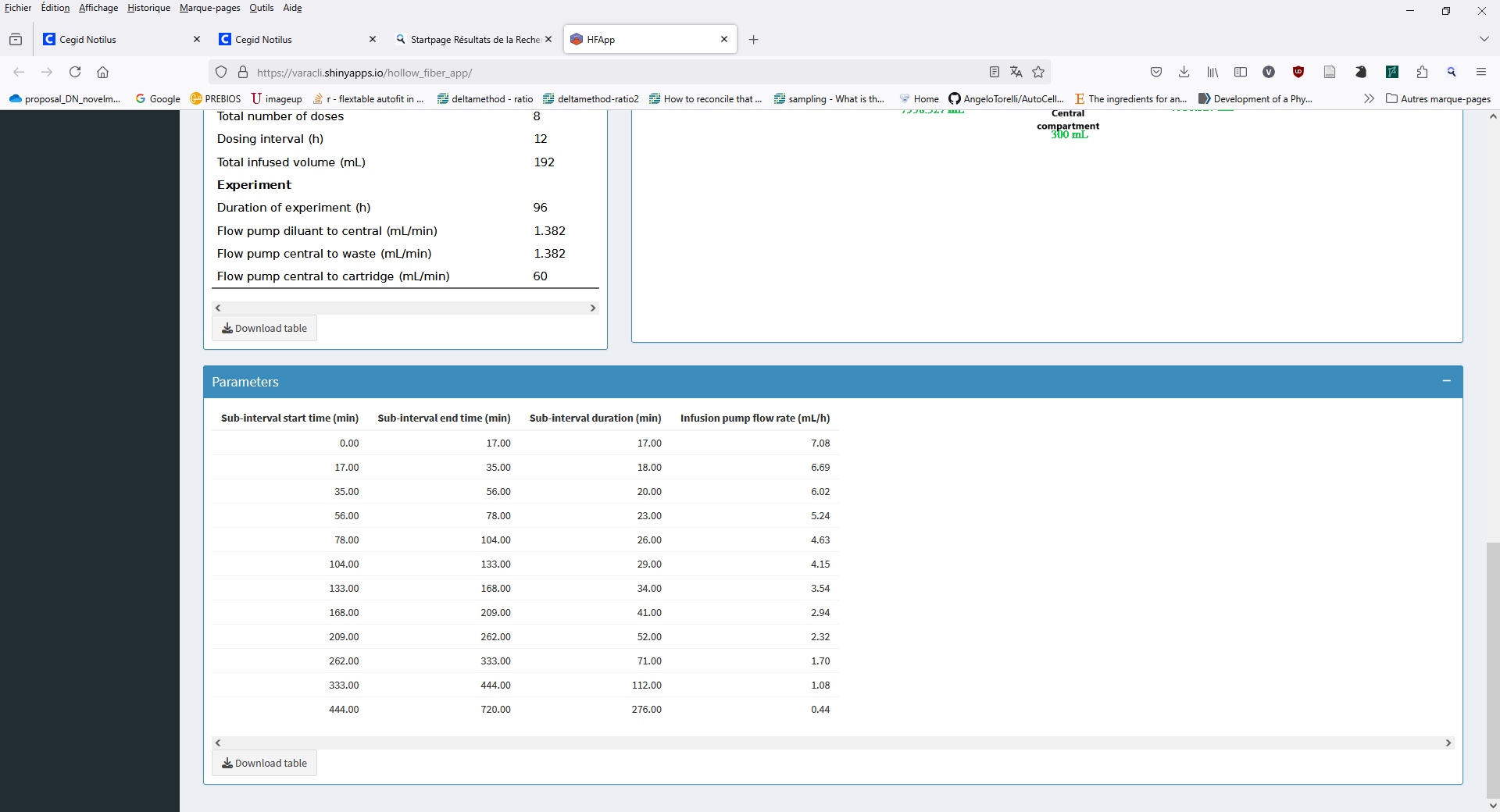


1. If you switch to the « simulation plotting » tab you will see a simulation of expected concentrations of linezolid. By hovering on the graph you will get more info.


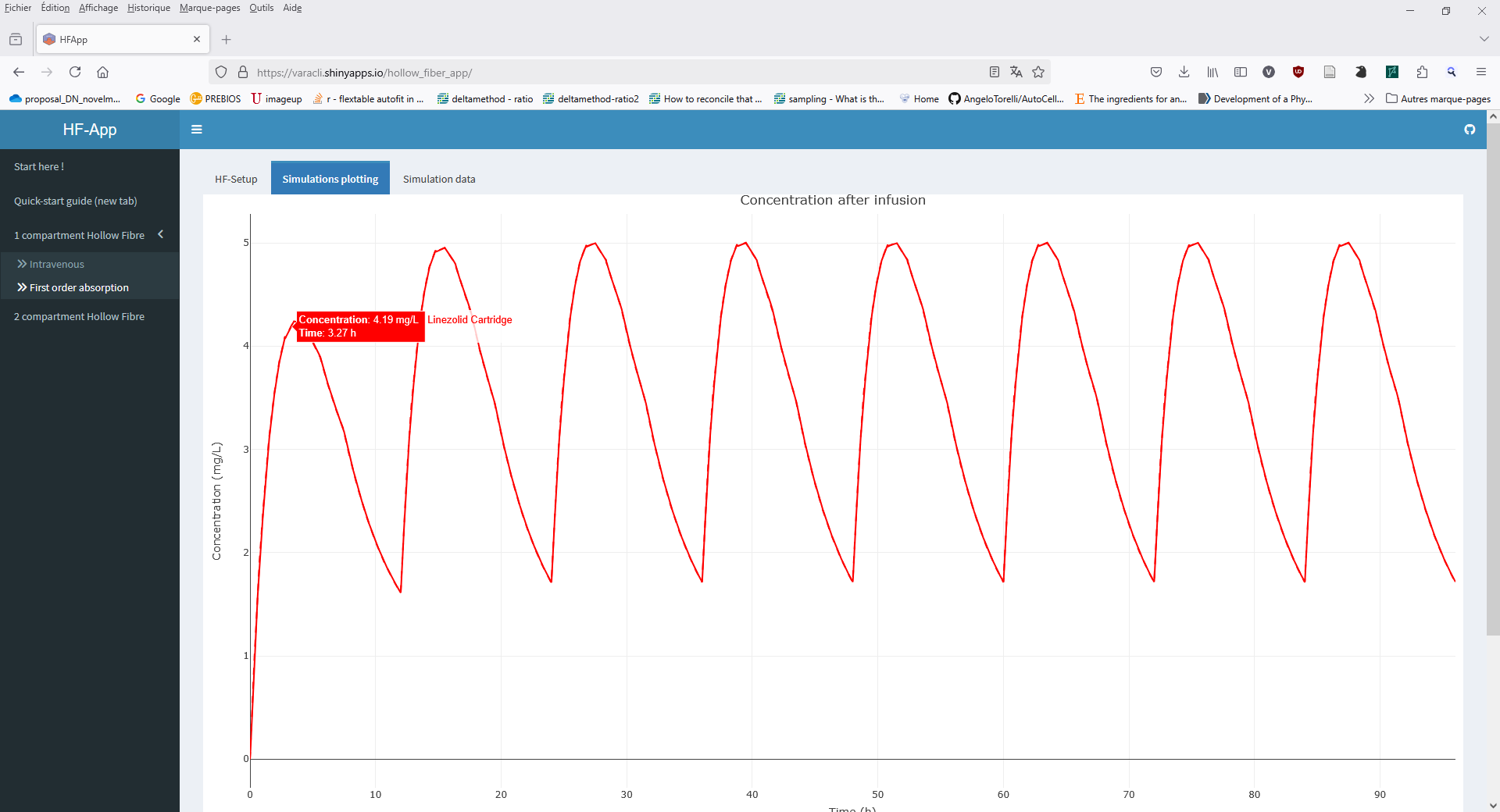


1. Lastly if you switch to the « Simulation data” tab you will get an excerpt of the simulated data points with an option to download the simulated data spreadsheet.


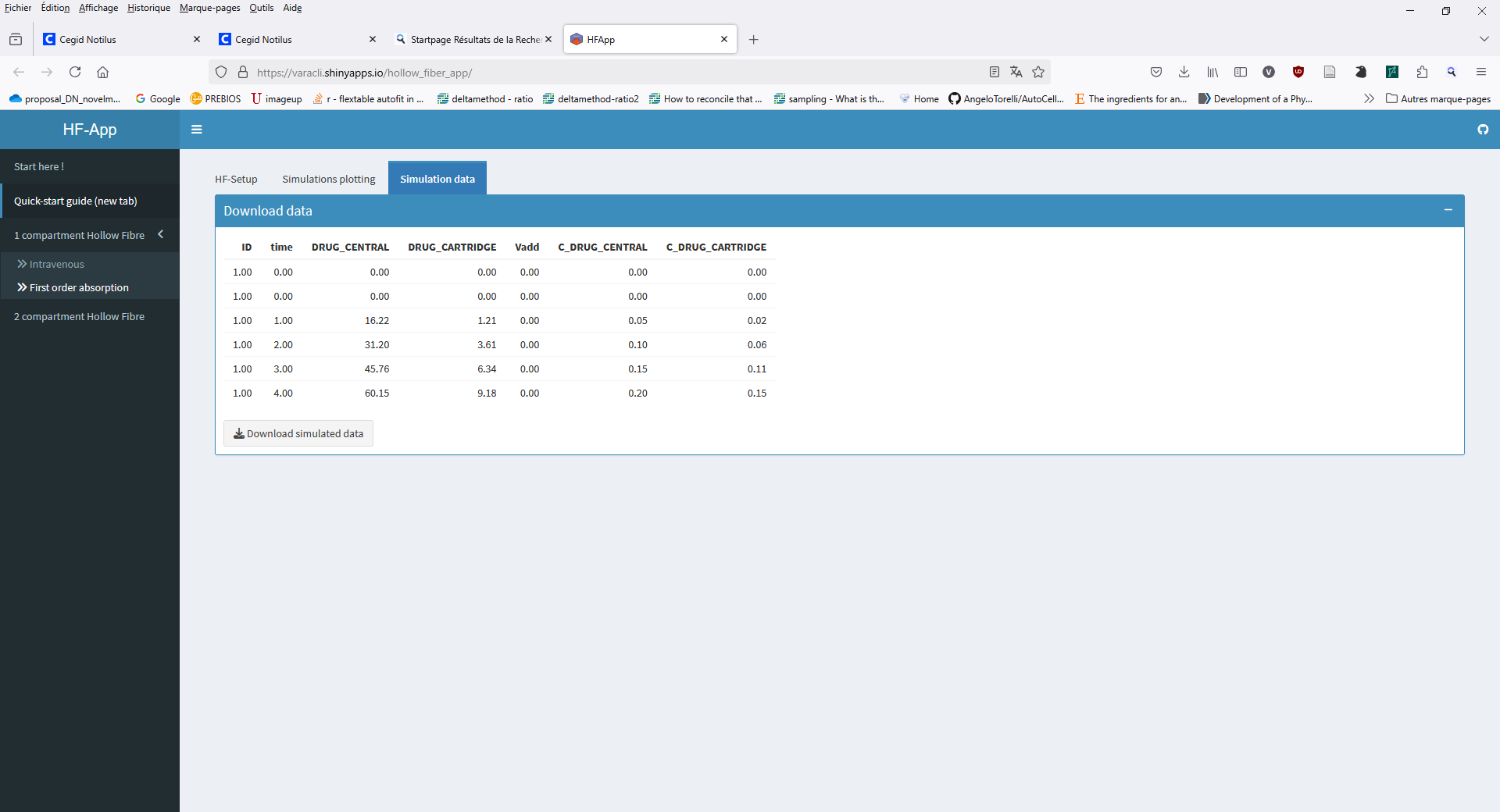


## Figure S1: Linezolid PK parameters computed from observations in the central reservoir and in the cartridge ECS from HFIM experiments reproducing plasma PK (top panels) and CSF PK (bottom panels) after administration of 900 mg q12 h (replicate number = 2-6).


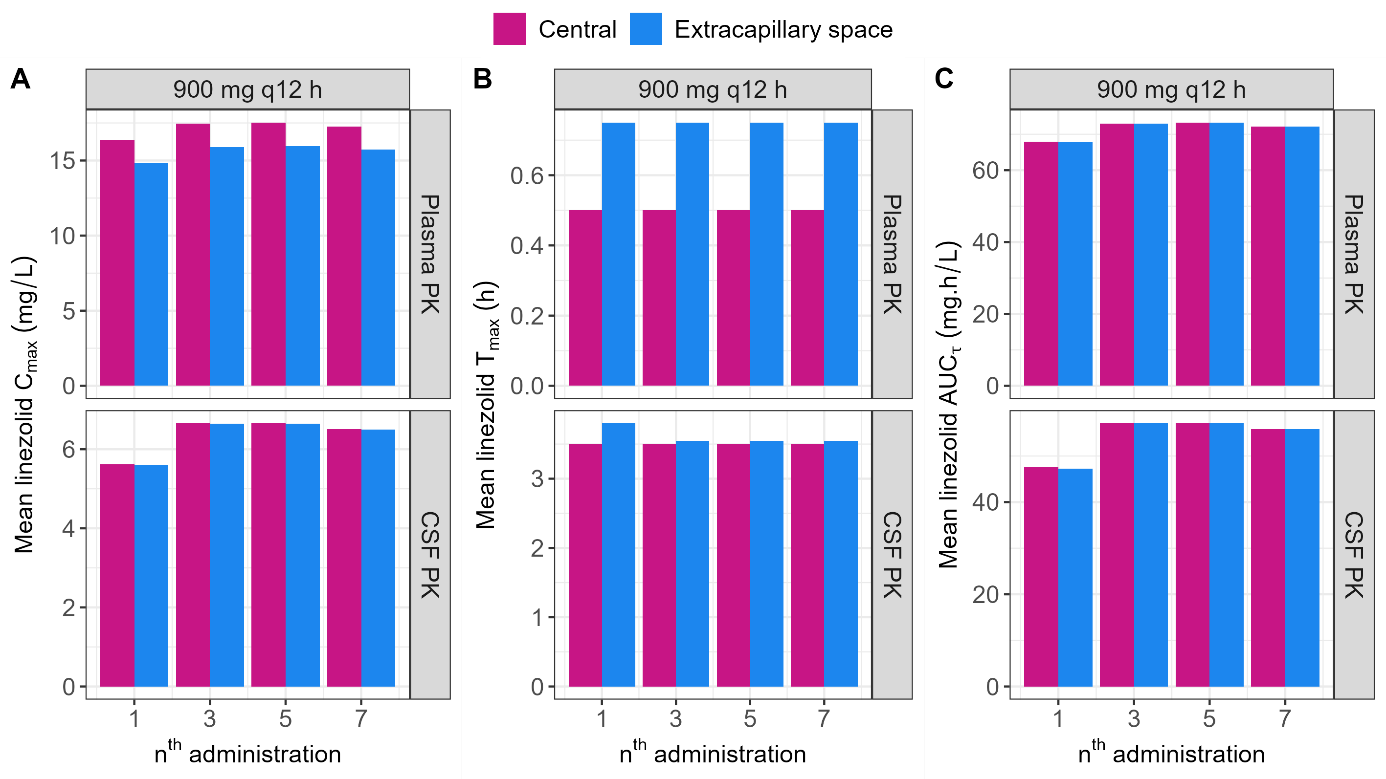


(A) Mean observed maximal concentrations (C*_max_*) of linezolid in the central reservoir (pink) and cartridge ECS (blue) after the 1*^st^*, 3*^rd^*, 5*^th^* and 7*^th^* administration. (B) Mean observed time to reach C*_max_* (T*_max_*) of linezolid in the central reservoir (pink) and cartridge ECS (blue) after the 1^st^, 3^rd^, 5^th^ and 7^th^ administration. (C) Mean observed area under the curve over the dosing interval (AUC_τ_) of linezolid in the central reservoir (pink) and cartridge ECS (blue) after the 1^st^, 3^rd^, 5^th^ and 7^th^ administration.

## Figure S2: Linezolid PK parameters computed from observations in the central reservoir and in the cartridge ECS from HFIM experiments reproducing plasma PK (top panels) and CSF PK (bottom panels) after administration of 900 mg q8 h (replicate number = 2).


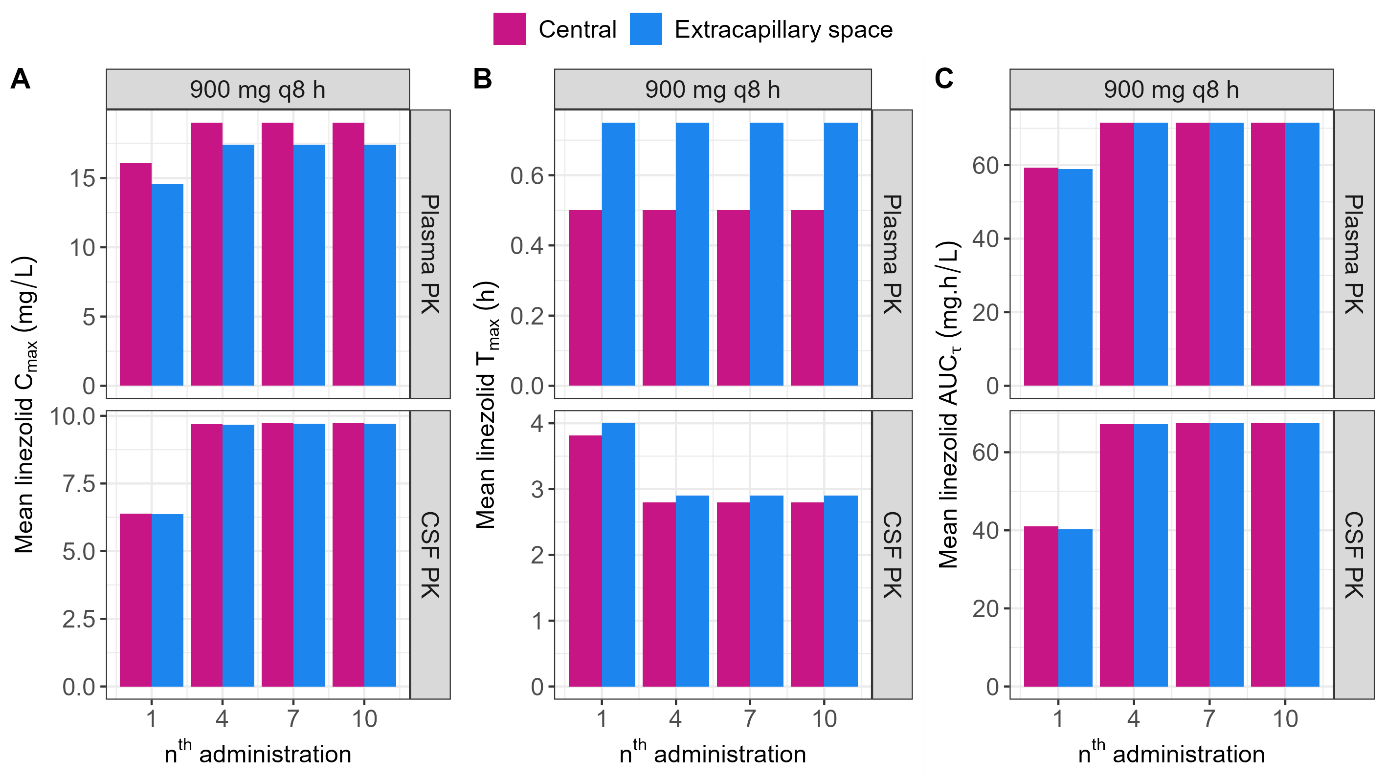


(A) Mean observed maximal concentrations (C*_max_*) of linezolid in the central reservoir (pink) and cartridge ECS (blue) after the 1*^st^*, 4*^th^*, 7*^th^* and 10*^th^* administration. (B) Mean observed time to reach C*_max_* (T*_max_*) of linezolid in the central reservoir (pink) and cartridge ECS (blue) after the 1*^st^*, 4*^th^*, 7*^th^* and 10*^th^* administration. (C) Mean observed area under the curve over the dosing interval (AUC_τ_) of linezolid in the central reservoir (pink) and cartridge ECS (blue) after the 1*^st^*, 4*^th^*, 7*^th^* and 10*^th^* administration.

## Figure S3: Target linezolid CSF concentrations from linezolid clinical population PK model (13) versus expected linezolid concentrations in the cartridge ECS for the first dose of 900 mg q12 h and 900 mg q8 h dosing regimen.


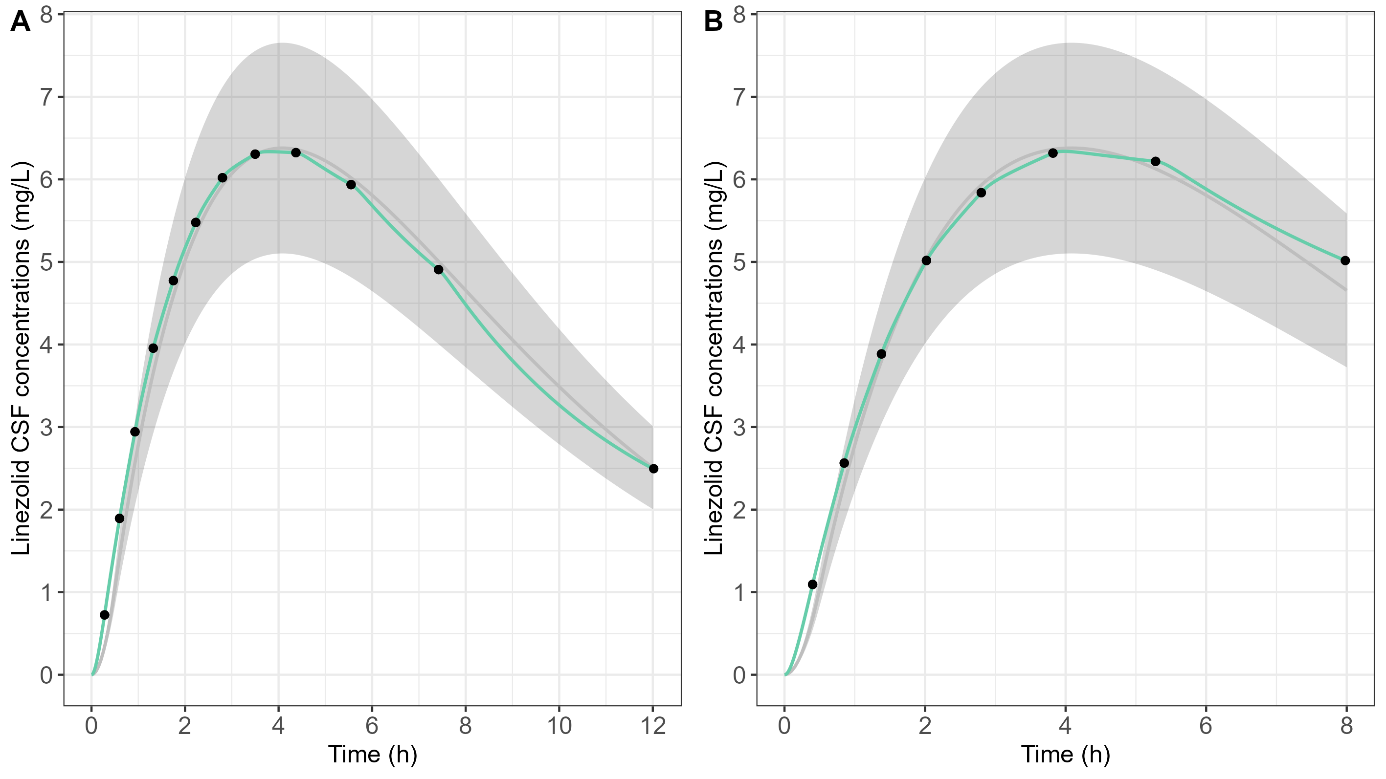


(A) First dose of 900 mg q12 h. (B) First dose of 900 mg q8 h.

The grey line corresponds to target linezolid concentrations. The grey area corresponds to a bias of 20% from the target linezolid concentrations. The green line corresponds to expected linezolid concentrations in the cartridge ECS with the infusion pump program shown in **Table S3**. Black points correspond to the expected concentrations at the end of each infusion sub-interval.

## Table S4: Bias and imprecision of the algorithm

| **Dosing regimen** | **MPE* (%)** | **RMSE** (mg/L)** |
| --- | --- | --- |
| 600 mg q 12 h | -0.461 | 0.0408 |
| 900 mg q 12 h | -3.90 | 0.181 |
| 900 mg q 8 h | -10.2 | 0.774 |

*Mean Percentage Error
**Root Mean Squared Error

The downward bias was low enough for us to justify using the simpler experimental setup rather than adding an extra reservoir. Bias and imprecision are higher for 900 mg q8 h dosing regimen which was expected since we were limited to using 8 sub-intervals (instead of 12 for the other dosing regimens).
